# The associations of unsweetened, sugar-sweetened, and artificially sweetened tea consumption with all-cause and cause-specific mortality in 195,361 UK Biobank participants: a large prospective cohort study

**DOI:** 10.3389/fnut.2025.1649279

**Published:** 2025-07-31

**Authors:** Hao Huang, Lei Zhang, Ding Zhang, Miaomiao Yang, Ying Lin, Zhiyong Wang, Pei Wei, Jiaqi Lin, Jingyao Huang, Pengfei Wei, Yinggang Chen, Baochang He, Ming Zhang, Dongsheng Hu, Fulan Hu

**Affiliations:** ^1^Department of Preventive Medicine, Zhuhai Campus of Zunyi Medical University, Zhuhai, Guangdong, China; ^2^Department of Epidemiology, School of Public Health, Nantong University, Nantong, Jiangsu, China; ^3^Department of Biostatistics and Epidemiology, School of Public Health, Shenzhen University Medical School, Shenzhen, Guangdong, China; ^4^Department of Biostatistics and Epidemiology, School of Public Health, Fujian Medical University, Fuzhou, Fujian, China; ^5^Department of Immunology, Zhuhai Campus of Zunyi Medical University, Zhuhai, Guangdong, China; ^6^Department of Endocrinology, Shenzhen University General Hospital, Shenzhen, Guangdong, China; ^7^Department of Gastrointestinal Surgery, Shenzhen Hospital, National Cancer Center/Cancer Hospital, Chinese Academy of Medical Sciences and Peking Union Medical College, Shenzhen, Guangdong, China

**Keywords:** tea, sweeteners, risk factor, mortality, UK Biobank

## Abstract

**Background:**

Tea consumption has been associations with a lower risk of mortality and numerous health benefits. However, it is still unclear whether consuming tea with or without sugar or sweeteners has different effects on mortality. It is necessary to investigate the associations of unsweetened, sugar-sweetened, and artificially sweetened tea consumption with all-cause mortality and cause-specific mortality.

**Methods:**

In this population-based cohort study of 195,361 UK Biobank participants who completed at least one 24-h dietary recall, we examined tea consumption by type (unsweetened, sugar-sweetened, artificially sweetened). Cox proportional hazards models and restricted cubic splines were used to assess nonlinear associations between tea intake and the risks of all-cause, cancer, and cardiovascular disease (CVD) mortality. We also conducted subgroup analyses stratified by genetic score for caffeine metabolism.

**Results:**

After a median follow-up of 13.6 years, 11,718 all-cause deaths were recorded, including 2,202 deaths from CVD and 6,415 from cancer. A U-shaped association was observed between tea consumption and mortality risk. Compared with non-consumers, individuals consuming 3.5–4.5 drinks per day of unsweetened tea had the lowest risks of all-cause (HR, 0.80; 95% CI: 0.75–0.86), cancer (HR, 0.86; 95% CI: 0.77–0.97), and CVD (HR, 0.73; 95% CI: 0.60–0.89). Sugar-sweetened tea showed no consistent or statistically significant associations with all-cause, cancer, or CVD mortality across different levels of consumption. Similarly, no significant associations were found for artificially sweetened tea. The observed associations between tea consumption and mortality were not modified by genetic predisposition to caffeine metabolism.

**Conclusion:**

Unsweetened tea consumption was significantly associated with a lower risk of all-cause, cancer, and CVD mortality. No consistent or statistically significant associations were observed for sugar-sweetened or artificially sweetened tea. The potential attenuation of tea’s protective effects by added sugar or artificial sweeteners warrants further investigation. Given current evidence, it may be advisable to consume tea without added sweeteners to optimize health benefits and longevity.

## 1 Introduction

Tea is one of the most widely consumed beverages globally, with an estimated 2 billion people drinking tea every day and per capita annual consumption exceeding 100 liters in countries such as the UK and China (1). Tea has been linked to potential health benefits, including antioxidant properties and protective effects against chronic diseases such as cardiovascular disease and certain cancers (2). Previous observational studies have shown that moderate tea consumption is associated with a reduced risk of mortality and other common causes of death (3, 4). However, the associations between different tea types and sweetening practices with long-term health outcomes remain unclear, particularly as tea is increasingly consumed with added sugar or artificial sweeteners.

In recent years, the consumption of sugar-sweetened beverages (SSBs) has increased worldwide, leading to a significant rise in added sugar intake (5). Epidemiological studies have extensively investigated the negative health effects of excessive sugar consumption, particularly from SSBs, including obesity, type 2 diabetes, cardiovascular diseases (CVD), and cancer (6–8). Conversely, artificially sweetened beverages have been suggested as a healthier alternative to SSBs due to their lower calorie content (9). However, concerns have been raised regarding the potential adverse effects of artificial sweeteners on metabolic health, as well as their association with increased mortality risk (10, 11). In fact, the World Health Organization (WHO) classified aspartame, a commonly used artificial sweetener, as possibly carcinogenic to humans in 2023 (12).

Given the popularity of sugar-sweetened and artificially sweetened teas and the limited evidence on their impact on health outcomes, further studies are needed to evaluate the relationships between tea consumption and long-term health risks. Therefore, this study aims to investigate the associations between sugar-sweetened, artificially sweetened, and unsweetened tea consumption and all-cause and cause-specific mortality by utilizing data from large-scale prospective cohort studies of the UK Biobank.

## 2 Materials and methods

### 2.1 Study design and population

UK Biobank is a large-scale multicenter prospective cohort study with more than 500,000 participants aged 37–73 years who were recruited between 2006 and 2010. The participants were primarily drawn from the general populations of 22 dedicated assessment centers in England, Wales, and Scotland. Participants provided information on health-related aspects through touchscreen questionnaires, verbal interviews, and physical measurements (13). The details of the study design and methods have been described in previous studies (14). Written informed consent was obtained from all study participants in accordance with the Declaration of Helsinki. Ethical approval for this study was granted by the National Health Service (NHS) North West Multicenter Research Ethics Committee. The study was conducted under application No.81680.

**Figure 1:**
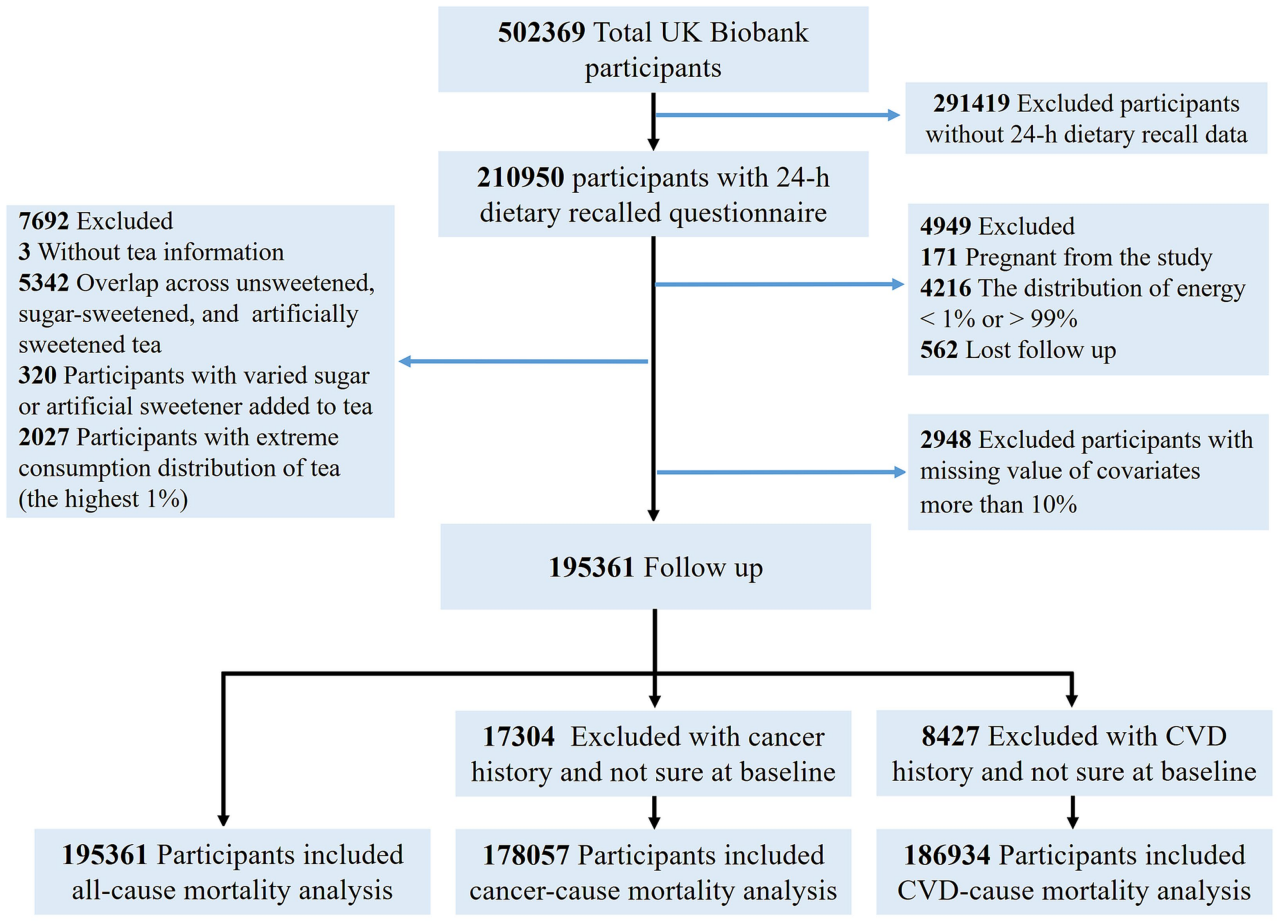
Flow chart of participant enrollment. CVD, cardiovascular disease.

In this study, we focused on participants who completed the online 24-h recall questionnaire at least once between April 2009 and June 2012. Participants were invited to complete the dietary recall on up to five separate occasions. The average dietary intake across all available recalls was used as an estimate of habitual intake. In total, 210,950 individuals had completed at least one dietary questionnaire. We excluded participants who were pregnant (*n* = 171), those with extreme energy intake (estimated energy requirement in the highest or lowest 1% of the distribution) (*n* = 4,216), individuals who were lost to follow-up (*n* = 562), or those with missing or overlapping information on tea consumption (*n* = 7,692). Additionally, we also excluded participants with more than two missing covariates (approximately 10%) (*n* = 2,948), leaving 195,361 participants for the primary analysis (Figure 1 and Supplementary Table S1).

### 2.2 Assessment of exposure

During the study, participants were asked about their tea consumption through a touchscreen questionnaire, “How many drinks of tea yesterday?” If participants reported drinking tea, they were asked to specify the type (standard tea, rooibos tea, green tea, herbal tea, or other tea) and to indicate whether they added sugar or sweeteners. Participants were categorized into four groups based on their tea consumption habits: non-consumers, unsweetened tea consumers, sugar-sweetened tea consumers, and artificially sweetened tea consumers. A tea consumer was defined as a participant who reported drinking tea at least once during the dietary recalls, based on responses to up to five 24-h dietary assessments. If participants consumed different types of tea (unsweetened, sugar-sweetened, or artificially sweetened tea) across the five recalls, they were classified as overlapping consumers and were subsequently excluded from the analysis (*n* = 5,342) (Supplementary Table S1). To minimize confusion regarding serving size (e.g., mug vs. cup), a standard drink size was defined as approximately 250 mL or 8.5 ounces. Tea consumption was categorized into five levels: 0–1.5, 1.5–2.5, 2.5–3.5, 3.5–4.5, and 4.5 or more drinks per day. Participants who reported drinking more than 10 cups of tea per day were asked to confirm their responses.

Data on potential confounding variables were obtained from baseline touchscreen questionnaires and health records, including sociodemographic characteristics, behavioral factors, and health conditions. Sociodemographic variables included age, sex, ethnicity (White or other), education level (college/university degree or other), and the Townsend Deprivation Index (TDI), a continuous measure of socioeconomic deprivation based on postcode, incorporating indicators such as unemployment, non-car ownership, non-home ownership, and household overcrowding. Behavioral factors included smoking status (never, former, current), pack-years of smoking (calculated as: [cigarettes per day ÷ 20] × years of smoking), alcohol consumption, and physical activity level (low, moderate, or high), assessed using the International Physical Activity Questionnaire (IPAQ) and categorized according to standard scoring protocols based on frequency, intensity, and duration of activity. Basal metabolic rate (BMR), reflecting resting energy expenditure, was calculated from anthropometric data and treated as a continuous variable. Dietary factors included intakes of total energy, total sugar, fruits, vegetables, red, and processed meats, coffee, milk, sugar-sweetened beverages (SSBs), artificially sweetened beverages (ASBs), and naturally sweet juices (NSJs). Beverage categories were defined as follows: sugar-sweetened drinks (e.g., carbonated soft drinks or fruit-flavored beverages), artificially sweetened beverages (e.g., squash, cordial, or fizzy drinks), and natural fruit and vegetable juices.

Health-related factors included body mass index (BMI, calculated as weight in kilograms divided by height in meters squared), self-rated overall health (poor, fair, good, or excellent), self-reported cardiovascular disease (myocardial infarction, angina, or stroke), self-reported cancer, family history of CVD or cancer, hypertension, diabetes, depression, long-standing illness, current use of cholesterol-lowering or antihypertensive medications, and use of vitamin or mineral supplements (including vitamins A, B, C, D, E, multivitamins, calcium, iron, zinc, selenium, and fish oil). Disease status was defined based on self-report, medication use, or hospital linkage using UK Biobank clinical classification codes.

### 2.3 Ascertainment of outcomes

Mortality data, including the date and cause of death, were obtained from the NHS Information Centre (for England and Wales) and the NHS Central Register (for Scotland). The underlying cause of death was classified using the International Statistical Classification of Diseases, 10th Revision (ICD-10). We examined all-cause mortality, cancer mortality (ICD-10 codes C00–D48), and cardiovascular disease (CVD) mortality (ICD-10 codes I00–I99). Participants were followed from the date of their first completed 24-h dietary recall until the date of death or the end of the follow-up period (March 11, 2023), whichever occurred first.

### 2.4 Genetic caffeine metabolism score

The UK Biobank genotyping was conducted using two genome-wide arrays: the UK BiLEVE array (for 50,000 participants) and the UK Biobank Axiom array (for 450,000 participants). These arrays were highly similar and collectively covered approximately 800,000 single-nucleotide polymorphisms (SNPs) and insertion–deletion markers (15). After excluding sample outliers based on heterozygosity and missingness, and applying kinship-based exclusions, genetic data on caffeine metabolism were available for 403,816 individuals in the analytic cohort (16, 17). We used four SNPs—rs2472297 (near CYP1A2), rs6968554 (near AHR), rs17685 (in POR), and rs56113850 (near CYP2A6)—to construct a genetic caffeine metabolism score (CMS_G4_), which has been shown to correlate with blood caffeine metabolite levels. These variants are located in or near genes involved in caffeine metabolism. A weighted CMS_G4_ was calculated by multiplying the number of risk alleles by their corresponding *β*-coefficients. The wCMS_G4_ was then divided into four categories: 0–2, >2–3, >3–4, and >4, with higher scores indicating faster caffeine metabolism.

### 2.5 Statistical analysis

Baseline characteristics related to tea consumption were presented as means with standard deviations (SDs) for continuous variables and as frequencies with percentages for categorical variables. We performed multiple imputations using chained equations to generate five datasets to address missing covariate data. Details of the missing data are summarized in Supplementary Table S2. The average amount of sugar or sweetener added to tea across multiple 24-h dietary recall questionnaires is presented in Supplementary Table S3. In addition, Spearman correlation coefficients were used to assess the consistency of tea consumption across multiple recalls (Supplementary Table S4).

Cox proportional hazard regression models were used to examine the association between tea consumption and the risk of all-cause and cause-specific mortality, with non-consumers serving as the reference group. Two models were constructed: a basic model and a multivariable-adjusted model. Model 1 was adjusted for baseline age (continuous) and sex (female or male). Model 2 was further adjusted for the following covariates: ethnicity (White or other), Townsend Deprivation Index (continuous), education level (degree or no degree), smoking status (current, former, or never), pack-years of smoking (continuous), overall health (poor, fair, good, or excellent), basal metabolic rate (continuous), physical activity level (low, moderate, or high), body mass index (continuous), hypertension (yes or no), diabetes (yes or no), depression (yes or no), family history of CVD disease (yes or no), family history of cancer (yes or no), long-standing illness (yes or no), use of cholesterol-lowering drug use or blood pressure drug use (yes or no), vitamin and mineral supplement use (yes or no), and dietary intakes of energy, total sugar, fresh fruit, vegetables, red and processed meats, alcohol, coffee, milk, naturally sweet juices, sugar-sweetened beverages, and artificially sweetened beverages. The proportional hazard assumption was tested using Schoenfeld residuals, and no significant violations were detected.

Restricted cubic spline (RCS) regression, a method for modeling non-linear relationships where the strength or direction of association may vary across the range of an independent variable, was used to assess potential non-linear associations between tea consumption and mortality outcomes. RCS models were fitted with four knots placed at the 5th, 35th, 65th, and 95th percentiles to evaluate non-linear relationships between the amounts of sugar or artificial sweeteners added to tea and risks of all-cause, CVD, and cancer mortality. Based on previous studies, individuals consuming five or more cups of tea per day were considered excessive drinkers. Tea consumption was classified into five categories: 0 to <1.5, 1.5 to <2.5, 2.5 to <3.5, 3.5 to <4.5, and ≥4.5 drinks per day. We conducted subgroup analyses to evaluate whether the associations between tea consumption and mortality differed by age (<60 or ≥60 years), sex (female or male), ethnicity (white or other), education (with or without degree), TDI (mean), BMI (<30 or ≥30 kg/m^2^), smoking status (former/current or never), alcohol consumption, coffee (mean), physical activity (low, moderate, or high), hypertension (yes or no), diabetes (yes or no), family history of CVD (yes or no), and family history of cancer (yes or no). To separately examine the effects of unsweetened, sugar-sweetened, and artificially sweetened tea, we performed stratified multivariable-adjusted models by wCMS_G4_ categories and by ethnicity (White vs. non-White). Given that standard black tea is the most commonly consumed type in the UK Biobank cohort, we also conducted a subgroup analysis restricted to standard tea consumers to assess consistency of associations (Supplementary Table S5).

In sensitivity analyses, we first excluded participants with missing covariates to assess the robustness of our findings. We then excluded deaths occurring within the first 2 years of follow-up to minimize potential reverse causality. Additionally, we excluded participants for whom added sugar in tea was excluded from total sugar intake calculations, and those who reported tea consumption in the past year but did not consume tea the day before the dietary recall. To evaluate potential confounding by overall diet quality, we further adjusted for a modified Alternative Healthy Eating Index (AHEI) score based on 11 components (Supplementary Table S6). As part of the E-value analysis, we estimated the strength of unmeasured confounding that would be required to explain away the observed associations between tea consumption and mortality. E-values and their lower confidence limits (LCLs) were calculated to support the sensitivity analysis. All analyses were performed using R software (version 4.1.3), and two-sided *p*-values <0.05 were considered statistically significant.

## 3 Results

### 3.1 Patients and characteristics

A total of 195,361 individuals were included in the analysis of all-cause mortality. For cause-specific mortality outcomes, participants without corresponding events were excluded, resulting in 186,934 participants for cardiovascular disease mortality and 178,057 for cancer mortality. Baseline characteristics of participants stratified by type of tea consumption are presented in Table 1. Among all participants, 162,516 (83.2%) were tea consumers. The majority consumed unsweetened tea (81.6%), followed by sugar-sweetened tea (12.2%) and artificially sweetened tea (6.2%). On average, 1.1 teaspoons of sugar and 1.4 teaspoons of artificial sweeteners were added per drink of tea. Compared to tea consumers, non-consumers tended to have a higher BMI and were more likely to prefer alcohol and coffee. Consumers of unsweetened tea were more likely to have higher educational attainment, lower BMI, lower basal metabolic rate, and generally exhibited healthier lifestyle behaviors. Consumers of sugar-sweetened tea were more likely to be male, with higher total energy intake and lower fruit consumption. Those who drank artificially sweetened tea were generally older, more likely to be current or former smokers, had higher BMI, were more frequently overweight, reported fair or poor self-rated health, and were more likely to have hypertension, diabetes, CVD, long-standing illness, and to use cholesterol-lowering or antihypertensive medications.

**Table 1:** Baseline characteristics of study participants by tea intake.

Characteristic	Total		Tea consumers		
		Nonconsumers	Unsweetened	Sugar-sweetened	Artificially sweetened
Participants, n	195,361	32,845	132,658	19,752	10,106
Mean Age (SD), y	56.1 (7.9)	55.0 (8.2)	56.3 (7.8)	55.7 (8.3)	57.4 (7.8)
Male, n (%)	87,473 (44.8)	15,867 (48.3)	54,394 (41.0)	12,382 (62.7)	4,830 (47.8)
Ethnicity, n (%)					
White	187,101 (96.1)	31,183 (95.3)	128,575 (97.2)	17,715 (89.9)	9,628 (95.4)
Other	7,693 (3.9)	1,545 (4.7)	3,704 (2.8)	1,985 (10.1)	459 (4.6)
Mean townsend deprivation index (SD)	−1.6 (2.9)	−1.3 (3.0)	−1.8 (2.8)	−1.2 (3.1)	−1.5 (2.9)
Education (%)					
Degree	84,068 (43.2)	12,798 (39.1)	62,035 (46.9)	6,398 (32.6)	2,837 (28.2)
No degree	110,542 (56.8)	19,909 (60.9)	70,201 (53.1)	13,219 (67.4)	7,213 (71.8)
Smoking status, n (%)					
Never	111,375 (57.1)	17,828 (54.4)	79,099 (59.7)	9,956 (50.5)	4,492 (44.5)
Former	68,758 (35.3)	11,313 (34.5)	46,063 (34.8)	6,837 (34.7)	4,545 (45.1)
Current	14,856 (7.6)	3,645 (11.1)	7,253 (5.5)	2,910 (14.8)	1,048 (10.4)
Mean pack-years of smoking for current or former smokers (SD)	20.6 (17.0)	23.9 (18.9)	18.2 (15.4)	23.7 (17.9)	25.6 (19.2)
Physical activity level, n (%)					
Low	30,698 (18.4)	5,750 (20.7)	19,798 (17.4)	3,336 (20.0)	1,814 (21.2)
Moderate	70,863 (42.5)	11,292 (40.6)	49,268 (43.3)	6,770 (40.6)	3,533 (41.4)
High	65,356 (39.2)	10,779 (38.7)	44,812 (39.4)	6,568 (39.4)	3,197 (37.4)
Mean BMI (SD), kg/m^2^	26.9 (4.6)	28.0 (5.2)	26.6 (4.5)	26.4 (4.1)	28.7 (4.9)
Mean basal metabolic rate (SD), KJ	6601.9 (1351.5)	6812.6 (1421.8)	6500.5 (1322.2)	6823.8 (1312.0)	6818.0 (1417.5)
Overall health, n (%)					
Poor	5,904 (3.0)	1,442 (4.4)	3,147 (2.4)	785 (4.0)	530 (5.3)
Fair	33,877 (17.4)	6,677 (20.4)	20,564 (15.5)	4,188 (21.3)	2,448 (24.3)
Good	116,517 (59.8)	18,709 (57.1)	80,377 (60.7)	11,663 (59.2)	5,768 (57.2)
Excellent	38,697 (19.8)	5,941 (18.1)	28,353 (21.4)	3,064 (15.6)	1,339 (13.3)
Hypertension, n (%)	43,568 (22.3)	7,651 (23.3)	28,584 (21.6)	4,304 (21.8)	3,029 (30.0)
Diabetes, n (%)	8,130 (4.2)	1,886 (5.8)	4,887 (3.7)	250 (1.3)	1,107 (11.0)
Depression, n (%)	3,884 (2.0)	688 (2.1)	2,608 (2.0)	394 (2.0)	194 (2.0)
Self-reported CVD, n (%)	8,233 (4.2)	1,574 (4.8)	4,894 (3.7)	992 (5.0)	773 (7.7)
Self-reported cancer, n (%)	17,048 (8.7)	2,620 (8.0)	11,872 (9.0)	1,591 (8.1)	965 (9.6)
Family history of CVD, n (%)	56,037 (29.1)	8,982 (27.9)	38,526 (29.4)	5,477 (28.2)	3,052 (30.8)
Family history of cancer, n (%)	52,451 (27.3)	8,491 (26.4)	36,057 (27.5)	5,216 (26.9)	2,687 (27.1)
Long-standing illness, n (%)	56,754 (29.6)	10,478 (32.5)	36,514 (28.0)	5,818 (30.1)	3,944 (39.9)
Number of long-term conditions, n (%)					
None	56,599 (29.0)	9,289 (28.3)	39,465 (29.7)	5,896 (29.9)	1,949 (19.3)
One	62,763 (32.1)	10,262 (31.2)	43,293 (32.6)	6,356 (32.2)	2,852 (28.2)
Two	41,544 (21.3)	7,055 (21.5)	27,890 (21.0)	4,130 (20.9)	2,469 (24.4)
Three and more	34,455 (17.6)	6,239 (19.0)	22,010 (16.6)	3,370 (17.1)	2,836 (28.1)
Cholesterol-lowering drug use, n (%)	29,902 (15.4)	5,647 (17.3)	18,658 (14.1)	2,938 (14.9)	2,659 (26.4)
Blood pressure drug use, n (%)	17,811 (9.1)	2,964 (9.1)	11,825 (8.9)	1,836 (9.3)	1,186 (11.8)
Vitamin and mineral supplement use, n (%)	63,443 (32.5)	9,971 (30.4)	43,917 (33.2)	5,959 (30.3)	3,596 (35.7)
Mean intake (SD)					
Energy, kcal/d	2098.6 (563.5)	2071.5 (594.0)	2084.9 (544.3)	2243.1 (604.1)	2085.0 (585.3)
Total sugar, g/d	119.9 (46.9)	116.8 (51.3)	117.5 (44.1)	142.2 (51.0)	116.6 (48.3)
Fruit, servings/d	3.5 (2.7)	3.0 (2.7)	3.7 (2.7)	2.6 (2.4)	3.2 (2.7)
Vegetables, servings/d	5.8 (4.2)	4.9 (4.0)	6.2 (4.2)	4.9 (3.9)	5.4 (4.2)
Red meat, servings/d	0.6 (0.7)	0.6 (0.7)	0.6 (0.7)	0.6 (0.8)	0.6 (0.7)
Processed meat, servings/d	1.0 (1.5)	1.0 (1.5)	1.0 (1.4)	1.1 (1.6)	1.1 (1.5)
Alcohol, g/d	16.3 (21.1)	18.0 (24.1)	15.9 (20.0)	16.7 (22.8)	15.4 (21.7)
Coffee, drinks/d	2.1 (1.8)	3.1 (2.0)	1.9 (1.6)	1.5 (1.6)	1.7 (1.7)
SSBs, drinks/d	0.5 (0.9)	0.6 (1.1)	0.4 (0.8)	0.6 (1.0)	0.6 (1.0)
ASBs, drinks/d	0.3 (0.8)	0.5 (1.0)	0.3 (0.7)	0.2 (0.6)	0.5 (0.9)
NSJs, drinks/d	0.6 (0.8)	0.6 (0.8)	0.6 (0.8)	0.6 (0.8)	0.5 (0.7)
Milk, drinks/d	0.1 (0.3)	0.1 (0.4)	0.1 (0.3)	0.1 (0.4)	0.1 (0.3)
Tea on average in past year, drinks/d	3.2 (2.7)	0.5 (1.4)	3.8 (2.6)	3.8 (2.6)	3.9 (2.6)
Tea, drinks/d					
Total tea	2.8 (1.9)	–	3.5 (1.6)	3.2 (1.5)	3.3 (1.5)
Standard tea	2.3 (1.8)	–	2.7 (1.7)	2.9 (1.5)	2.9 (1.6)
Rooibos tea	0.1 (0.5)	–	0.1 (0.5)	0.0 (0.3)	0.1 (0.4)
Green tea	0.2 (0.6)	–	0.2 (0.7)	0.1 (0.3)	0.1 (0.4)
Herbal tea	0.2 (0.7)	–	0.3 (0.7)	0.1 (0.4)	0.1 (0.5)
Other tea	0.1 (0.5)	–	0.1 (0.6)	0.1 (0.5)	0.1 (0.6)
Type of sweetener added to tea, teaspoons/d					
Sugar	0.1 (0.4)	–	–	1.1 (0.6)	–
Artificial sweetener	0.1 (0.3)	–	–	–	1.4 (0.6)
Mean AHEI^*^ (SD)	52.2 (13.4)	49.2 (13.4)	53.8 (13.2)	47.2 (12.7)	50.0 (12.8)
Mean completed 24-h dietary recalls (SD), n	2.2 (1.2)	2.0 (1.1)	2.3 (1.2)	2.0 (1.1)	2.0 (1.1)
Median wCMS_G4_ (IQR)	3.7 (2.6–4.9)	3.7 (2.6–4.9)	3.8 (2.6–5.0)	3.7 (2.6–4.9)	3.8 (2.6–5.0)

^*^AHEI, The modified Alternative Healthy Eating Index is a combination of nine dietary indicators, including vegetables, fruit, whole grains, sugar-sweetened beverages and fruit juice, nuts and legumes, red or processed meat, Trans fat, long-chain (n-3) fatty acids (EPA + DHA), polyunsaturated fat intake (% of energy), sodium, and alcohol. The scores were summed up producing an overall score ranging from 0 to 110. BMI, body mass index; CVD, cardiovascular disease; SSBs, sugar-sweetened beverages; ASBs, artificially sweetened beverages; NSJs, naturally sweet juices. CMSG4, genetic caffeine metabolism scores. One drink is equal to approximately 250 mL or 8.5 ounces.

### 3.2 Associations between tea consumption and all-cause mortality

**Figure 2:**
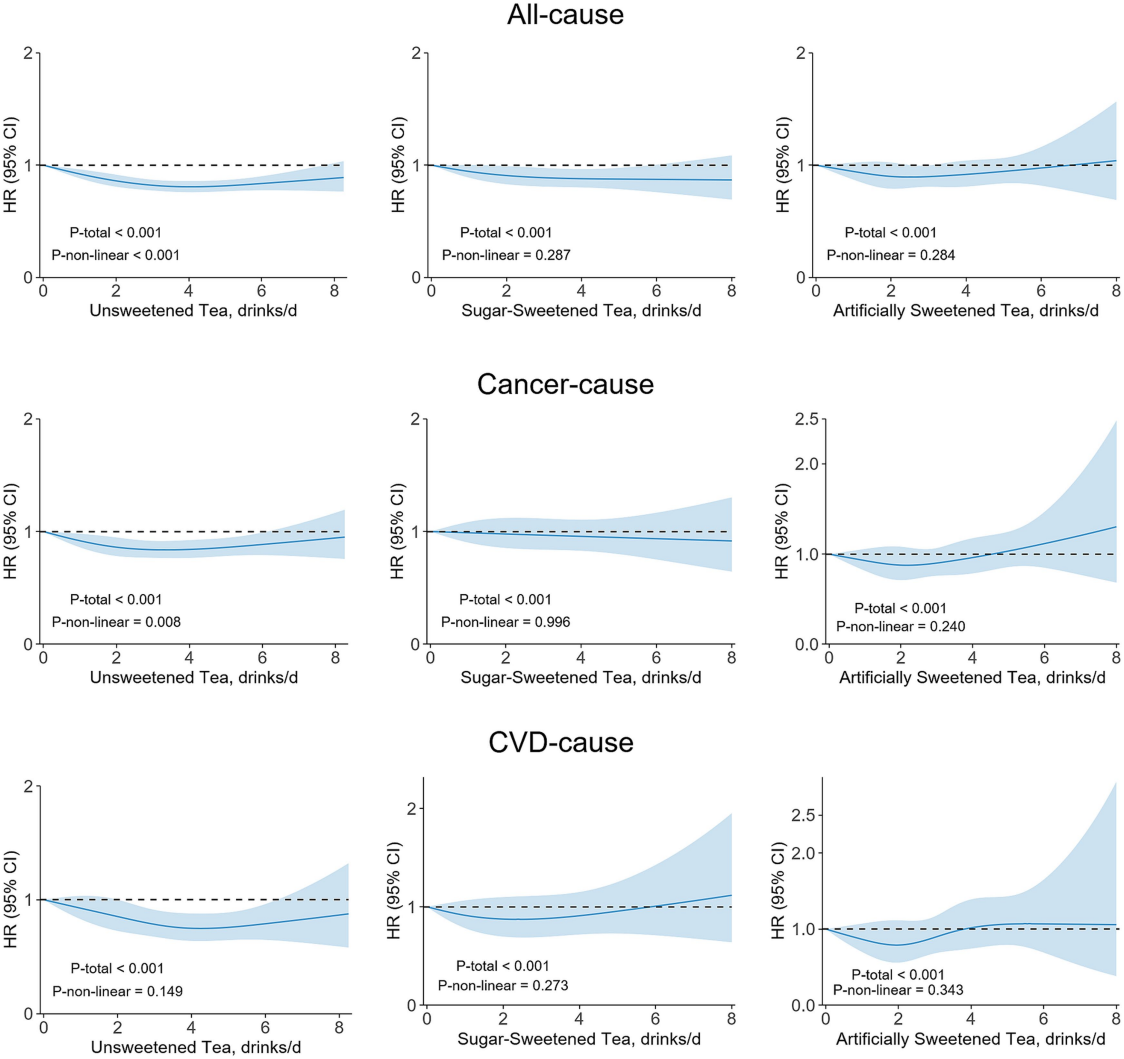
Dose–response associations of tea consumption with all-cause (top), cancer (middle), and CVD (bottom) mortality. CVD, cardiovascular disease; HR, hazard ratio. CVD, cardiovascular disease. One drink is equal to approximately 250 mL or 8.5 ounces. Multivariable Cox regression model with restricted cubic splines adjusted for age (continuous), gender, Townsend deprivation index (continuous), education level (degree or no degree), ethnicity (white or other), smoking status (current, former, or never), pack-years of smoking (continuous), Overall health (poor, fair, good, or excellent), Basal metabolic rate (continuous), physical activity level (low, moderate, or high), body mass index (continuous), hypertension (yes or no), diabetes (yes or no), depression (yes or no), family history of CVD disease (yes or no), family history of cancer (yes or no), long-standing illness (yes or no), cholesterol-lowering drug use (yes or no), blood pressure drug use (yes or no), vitamin and mineral supplement (yes or no), and intake of energy, total sugar, fresh fruit, vegetables, red meat, processed meat, alcohol, coffee, milk, naturally sweet juices, sugar-sweetened beverages, and artificially sweetened beverages.

Over a median follow-up of 13.6 years (IQR 13.0–14.4 years), 11,718 deaths from all causes were recorded, including 6,415 cancer deaths and 2,202 CVD deaths. Cox proportional hazards models revealed a significant inverse dose-dependent association between tea consumption and all-cause mortality (*p* < 0.001) after adjustment for multiple covariates (Table 2). Compared with non-consumers, participants who consumed unsweetened tea had progressively lower risks of all-cause mortality: 0–1.5 drinks (HR = 0.92; 95% CI: 0.85, 0.99), 1.5–2.5 drinks (HR = 0.83; 95% CI: 0.78, 0.89), 2.5–3.5 drinks (HR = 0.84; 95% CI: 0.78, 0.90), 3.5–4.5 drinks (HR = 0.80; 95% CI: 0.75, 0.86), and >4.5 drinks (HR = 0.83; 95% CI: 0.77, 0.89), respectively. For sugar-sweetened tea, hazard ratios for all-cause mortality across increasing consumption categories were 0.81 (95% CI, 0.70–0.94), 0.95 (95% CI, 0.84–1.07), 0.91 (95% CI, 0.80–1.03), 0.85 (95% CI, 0.74–0.98), and 0.87 (95% CI, 0.76–0.99), respectively (Table 2). The inverse association between unsweetened tea consumption and all-cause mortality was confirmed in RCS models, showing a significant U-shaped non-linear relationship (*P* for overall association <0.001; *P* for non-linearity <0.001) (Figure 2). In contrast, a monotonically decreasing, linear relationship was observed for sugar-sweetened tea (*P* for non-linearity >0.05). However, no significant association was observed between artificially sweetened tea and all-cause mortality in either the multivariable-adjusted Cox model or the RCS analysis.

**Table 2:** Associations of tea consumption with risk of all-cause and cause-specific mortality.

Outcome	Nonconsumers	Tea consumers				
		0–1.5 drinks/d	1.5–2.5 drinks/d	2.5–3.5 drinks/d	3.5–4.5 drinks/d	>4.5 drinks/d
Unsweetened tea						
All-cause mortality (*n* = 165,503)						
Events, n (%)	2,286 (7.0)	946 (5.7)	1,493 (5.4)	1,593 (5.4)	1,333 (5.2)	1,752 (5.3)
Basic model^†^	1 (Reference)	0.75 (0.70–0.81)	0.68 (0.64–0.73)	0.69 (0.65–0.74)	0.68 (0.63–0.72)	0.72 (0.68–0.77)
Multivariable model^*^	1 (Reference)	0.92 (0.85–0.99)	0.83 (0.78–0.89)	0.84 (0.78–0.90)	0.80 (0.75–0.86)	0.83 (0.77–0.89)
Cancer mortality (*n* = 150,807)						
Events, n (%)	941 (3.1)	401 (2.6)	668 (2.6)	658 (2.5)	606 (2.6)	774 (2.6)
Basic model^†^	1 (Reference)	0.78 (0.70–0.88)	0.75 (0.68–0.83)	0.70 (0.64–0.78)	0.75 (0.68–0.83)	0.78 (0.71–0.85)
Multivariable model^*^	1 (Reference)	0.90 (0.80–1.01)	0.87 (0.78–0.96)	0.81 (0.73–0.90)	0.86 (0.77–0.97)	0.87 (0.78–0.97)
CVD mortality (*n* = 158,877)						
Events, n (%)	350 (1.1)	147 (0.9)	213 (0.8)	244 (0.9)	179 (0.7)	228 (0.7)
Basic model^†^	1 (Reference)	0.74 (0.61–0.90)	0.62 (0.52–0.74)	0.68 (0.58–0.80)	0.59 (0.49–0.70)	0.62 (0.53–0.73)
Multivariable model^*^	1 (Reference)	0.93 (0.76–1.13)	0.78 (0.66–0.93)	0.85 (0.72–1.02)	0.73 (0.60–0.89)	0.75 (0.62–0.91)
Sugar-sweetened tea						
All-cause mortality (*n* = 52,597)						
Events, n (%)	2,286 (7.0)	192 (5.9)	326 (6.9)	310 (7.1)	251 (7.1)	339 (8.6)
Basic model^†^	1 (Reference)	0.78 (0.68–0.91)	0.90 (0.81–1.02)	0.89 (0.79–1.01)	0.88 (0.77–1.00)	1.04 (0.93–1.16)
Multivariable model^*^	1 (Reference)	0.81 (0.70–0.94)	0.95 (0.84–1.07)	0.91 (0.80–1.03)	0.85 (0.74–0.98)	0.87 (0.76–0.99)
Cancer mortality (*n* = 48,303)						
Events, n (%)	941 (3.1)	80 (2.7)	144 (3.3)	139 (3.4)	108 (3.4)	136 (3.8)
Basic model^†^	1 (Reference)	0.81 (0.65–1.02)	1.00 (0.84–1.19)	0.98 (0.82–1.18)	0.95 (0.78–1.16)	1.05 (0.88–1.26)
Multivariable model^*^	1 (Reference)	0.84 (0.66–1.06)	1.02 (0.85–1.23)	1.01 (0.82–1.21)	0.94 (0.76–1.17)	0.93 (0.75–1.14)
CVD mortality (*n* = 49,977)						
Events, n (%)	350 (1.1)	35 (1.1)	49 (1.1)	47 (1.1)	35 (1.1)	54 (1.5)
Basic model^†^	1 (Reference)	0.86 (0.60–1.21)	0.83 (0.61–1.12)	0.82 (0.61–1.12)	0.75 (0.53–1.07)	1.02 (0.76–1.36)
Multivariable model^*^	1 (Reference)	0.92 (0.64–1.31)	0.94 (0.69–1.29)	0.91 (0.66–1.26)	0.83 (0.57–1.21)	0.95 (0.68–1.33)
Artificially sweetened tea						
All-cause mortality (*n* = 42,951)						
Events, n (%)	2,286 (7.0)	125 (8.4)	187 (8.3)	204 (9.2)	164 (8.6)	217 (9.7)
Basic model^†^	1 (Reference)	1.00 (0.84–1.20)	0.97 (0.84–1.13)	1.05 (0.91–1.21)	1.03 (0.88–1.20)	1.14 (0.99–1.31)
Multivariable model^*^	1 (Reference)	0.91 (0.76–1.10)	0.89 (0.76–1.03)	0.94 (0.81–1.09)	0.90 (0.77–1.07)	0.94 (0.81–1.09)
Cancer mortality (*n* = 39,305)						
Events, n (%)	941 (3.1)	54 (3.9)	68 (3.3)	71 (3.6)	63 (3.7)	91 (4.5)
Basic model^†^	1 (Reference)	1.06 (0.81–1.40)	0.87 (0.68–1.11)	0.92 (0.72–1.17)	1.00 (0.77–1.29)	1.19 (0.96–1.48)
Multivariable model^*^	1 (Reference)	1.01 (0.77–1.33)	0.84 (0.66–1.08)	0.89 (0.69–1.14)	0.96 (0.74–1.25)	1.09 (0.86–1.37)
CVD mortality (*n* = 40,562)						
Events, n (%)	350 (1.1)	15 (1.1)	25 (1.2)	36 (1.8)	24 (1.4)	37 (1.8)
Basic model^†^	1 (Reference)	0.80 (0.48–1.34)	0.86 (0.58–1.30)	1.25 (0.89–1.76)	1.01 (0.67–1.52)	1.31 (0.93–1.84)
Multivariable model^*^	1 (Reference)	0.71 (0.42–1.19)	0.78 (0.51–1.17)	1.07 (0.75–1.53)	0.85 (0.55–1.30)	1.05 (0.73–1.52)

CVD, cardiovascular disease. One drink is equal to approximately 250 mL or 8.5 ounces. ^†^Basic model, estimates are hazard ratios (95% CIs) from Cox regression models adjusted for age (continuous) and sex. ^*^Multivariable model, estimates are hazard ratios (95% CIs) from multivariable Cox proportional hazard models adjusted for age (continuous), gender, Townsend deprivation index (continuous), education level (degree or no degree), ethnicity (white or other), smoking status (current, former, or never), pack-years of smoking (continuous), Overall health (poor, fair, good, or excellent), Basal metabolic rate (continuous), physical activity level (low, moderate, or high), body mass index (continuous), hypertension (yes or no), diabetes (yes or no), depression (yes or no), family history of CVD disease (yes or no), family history of cancer (yes or no), long-standing illness (yes or no), cholesterol-lowering drug use (yes or no), blood pressure drug use (yes or no), vitamin and mineral supplement (yes or no), and intake of energy, total sugar, fresh fruit, vegetables, red meat, processed meat, alcohol, coffee, milk, naturally sweet juices, sugar-sweetened beverages, and artificially sweetened beverages.

### 3.3 Associations between tea consumption and cause-specific mortality

In both multivariable Cox regression and nonlinear RCS models, we observed inverse associations between unsweetened tea consumption and mortality from cancer and CVD. For cancer-specific mortality, the HRs associated with daily unsweetened tea consumption were: 0–1.5 drinks (HR = 0.90; 95% CI: 0.80, 1.01), 1.5–2.5 drinks (HR = 0.87; 95% CI: 0.78, 0.96), 2.5–3.5 drinks (HR = 0.81; 95% CI: 0.73, 0.90), 3.5–4.5 drinks (HR = 0.86; 95% CI: 0.77, 0.97), and >4.5 drinks (HR = 0.87; 95% CI: 0.78, 0.97). For CVD-specific mortality, the corresponding HRs were: 0 to 1.5 drinks (HR = 0.93; 95% CI: 0.76, 1.13), 1.5–2.5 drinks (HR = 0.78; 95% CI: 0.66, 0.93), 2.5–3.5 drinks (HR = 0.85; 95% CI: 0.72, 1.02), 3.5–4.5 drinks (HR = 0.73; 95% CI: 0.60, 0.89), and >4.5 drinks (HR = 0.75; 95% CI: 0.62, 0.91), respectively. A nonlinear U-shaped relationship between unsweetened tea and cancer mortality was observed (*P* for nonlinearity =0.008). In contrast, a linear inverse association was observed between unsweetened tea and CVD mortality, with no evidence of nonlinearity (*P* for nonlinearity >0.05). For both sugar-sweetened and artificially sweetened tea, the number of cancer- and CVD-related deaths was relatively small. While potential associations were observed, they were not statistically significant. To further illustrate survival probabilities, Kaplan–Meier survival curves were generated for all-cause, cancer, and CVD mortality (Supplementary Figure S1). Participants with moderate intake of unsweetened tea demonstrated significantly better survival outcomes compared to non-consumers. These findings are consistent with the Cox model results.

### 3.4 Effect modification by genetic caffeine metabolism score

To examine whether the association between tea consumption and mortality was influenced by genetic variation in caffeine metabolism, we analyzed data stratified by weighted caffeine metabolism score (wCMS_G4_) derived from four established SNPs. Participants drank >4.5 drinks/d unsweetened tea had similar inverse associations with all-cause mortality in the lower strata of wCMS_G4_ 0–2 [HR = 0.73 (95% CI, 0.58–0.93)] to those in the higher strata of wCMS_G4_ >4 with >4.5 drinks/d [HR = 0.80 (95% CI, 0.71–0.90)], indicating no significant modification of the association between unsweetened tea and all-cause mortality by caffeine metabolism genotype (Supplementary Table S7). However, no statistically significant inverse association was observed for cancer mortality in any wCMS_G4_ subgroup among those consuming >4.5 drinks/day of unsweetened tea. The effect size of the association between >4.5 drinks/d unsweetened tea and CVD mortality was much lower in the strata of wCMS_G4_ 3–4 [HR = 0.69 (95%CI 0.49–0.99)] than that in the strata of wCMS_G4_ > 4 [HR = 0.71 (95%CI 0.53–0.97)] (Supplementary Tables S8, S9). For sugar-sweetened and artificially sweetened tea, no statistically significant associations with all-cause, cancer, or CVD mortality were observed in any genetic subgroup. Additionally, for white individuals consuming more than 4.5 drinks, we found similar inverse associations of unsweetened tea with all-cause mortality (HR = 0.77, 95% CI 0.60–0.98) in the lower strata of wCMS_G4_ 0–2 and in the higher strata of wCMS_G4_ > 4 (HR = 0.80, 95% CI 0.71–0.90). No evidence of effect modification by wCMS_G4_ was found for the associations between unsweetened tea and all-cause or cancer mortality in White individuals (Supplementary Tables S10–S12).

### 3.5 Subgroup analyses

In stratified analyses, the associations between tea consumption and all-cause or cause-specific mortality did not differ significantly by age group, sex, ethnicity, education, Townsend Deprivation Index (TDI), BMI, smoking status, alcohol intake, coffee intake, physical activity (IPAQ), hypertension, diabetes, family history of cancer, or family history of CVD. After adjustment for potential confounders, inverse associations between unsweetened tea consumption and all-cause mortality were observed consistently across most subgroups, except among participants of non-White ethnicity, those with low physical activity levels, and those with diabetes. A significant interaction was found between unsweetened tea consumption and coffee intake on all-cause mortality (*P* for interaction =0.03) (Supplementary Figure S2), with a stronger inverse association observed among participants with low coffee consumption compared to those with high coffee consumption. Inverse associations between sugar-sweetened tea and all-cause mortality were significant only in the following subgroups: Age ≥ 60 years, white ethnicity, BMI < 30 kg/m^2^, Alcohol intake ≥ average level, Presence of hypertension, No diabetes, and No family history of CVD. For cancer mortality, inverse associations with unsweetened tea were significant only among individuals with: TDI above the average level, BMI < 30 kg/m^2^, former or current smoking status, and No diabetes (Supplementary Figure S3). A significant interaction was observed between sugar-sweetened tea and hypertension on cancer mortality (*P* for interaction =0.02), with a stronger inverse association among individuals with hypertension than those without. For CVD mortality, we observed a multiplicative interaction between ethnicity and artificially sweetened tea (*P* for interaction =0.01), where the hazard ratio among White participants was much lower than that among non-White individuals. A similar interaction was identified between artificially sweetened tea and hypertension (*P* for interaction =0.04), with a stronger inverse association in participants without hypertension compared to those with hypertension (Supplementary Figure S4). To determine whether these associations were primarily driven by black tea, we conducted a subgroup analysis limited to participants who reported consuming standard tea. The results for all-cause, cancer, and CVD mortality were consistent with the overall findings, indicating that the observed inverse associations were largely attributable to black tea consumption (Supplementary Table S13).

### 3.6 Sensitivity analyses

Sensitivity analyses demonstrated that the observed associations were robust under multiple scenarios: Excluding participants with missing covariates did not materially alter the associations between tea consumption and mortality (Supplementary Table S14). Excluding deaths within the first 2 years of follow-up (Supplementary Table S15). Excluding added sugar in tea from the total sugar intake did not change the results (Supplementary Table S16). Removing participants who reported drinking tea in the past year but not on the recall day did not significantly alter the estimates (Supplementary Table S17). Additional adjustment for a modified AHEI score also yielded consistent results, supporting the robustness of our findings (Supplementary Table 18). Further adjustment for the total number of chronic conditions at baseline did not materially change the results, indicating robustness to baseline multimorbidity (Supplementary Table 19). Moreover, E-values for the association of unsweetened, sugar-sweetened, and artificially sweetened tea with all-cause mortality ranged from 1.29 (lower confidence limit [LCL], 1.00) to 1.81 (LCL, 1.60), suggesting a moderate level of unmeasured confounding would be required to fully explain these associations (Supplementary Table S20).

## 4 Discussion

In this large prospective cohort study using data from the UK Biobank, involving 195,361 participants, we found that moderate consumption of both unsweetened and sugar-sweetened tea was associated with a reduced risk of all-cause and cause-specific (cancer and CVD) mortality. Compared with non-consumers, those who consumed 3.5–4.5 drinks per day of unsweetened tea had a 20% lower risk of all-cause mortality. In addition, unsweetened tea consumption at the same level was associated with a 14% lower risk of cancer mortality and a 27% lower risk of CVD mortality. However, sugar-sweetened tea was not significantly associated with all-cause, cancer, or CVD mortality. Similarly, no significant associations were observed between artificially sweetened tea and the risk of all-cause or cause-specific mortality, potentially due to the adverse effects of artificial sweeteners.

Tea is among the most widely consumed beverages globally, particularly green and black tea (18). These beverages are rich in bioactive compounds, including polyphenols, catechins, and flavonoids, which exert anti-inflammatory, antioxidant, and anti-carcinogenic effects (19). Previous observational studies have consistently shown that moderate tea consumption has beneficial effects on cardiovascular health, including reduced risks of heart disease and stroke (20, 21). The bioactive constituents in tea—especially catechins and flavonoids—have been found to improve lipid profiles, lower blood pressure, and enhance endothelial function (22). Furthermore, several studies have indicated that tea consumption may reduce the risk of various cancers, including breast, ovarian, colorectal, and prostate cancer (23–26). The antioxidant properties of tea polyphenols are believed to inhibit tumor growth and contribute to a lower cancer risk (27). Meta-analyses of observational studies have also reported a significant association between tea intake and reduced all-cause mortality (4, 28, 29). These findings are reinforced by our current results, particularly for unsweetened tea, which consistently demonstrated the strongest inverse associations across mortality outcomes.

The global consumption of SSBs and ASBs has been increasing. However, recent studies have highlighted the potential adverse health effects associated with beverages containing added sugars or artificial sweeteners (30–32). In our study, we found a significant inverse association between unsweetened tea consumption and all-cause, CVD, and cancer mortality. In contrast, no significant associations were observed for sugar-sweetened or artificially sweetened tea, suggesting that the addition of sugar or artificial sweeteners may attenuate the health benefits of tea consumption. In addition, Mendelian randomization studies have been conducted to explore the causal relationship between tea consumption and mortality. Some of these studies, using genetic variants as instrumental variables, did not find a causal association between tea intake and reduced mortality risk (33, 34). One possible explanation is that such studies often do not account for modifiers such as added sugar or sweeteners, which may confound the associations. Moreover, Mendelian randomization analyses have inherent limitations, including the assumption that the genetic instruments are valid and the potential for horizontal pleiotropy. These limitations suggest that findings should be interpreted cautiously, and future studies should incorporate beverage composition, including sugar or sweetener content, to more accurately assess causal effects of tea consumption on mortality.

The intake of added sugars, such as sucrose and high-fructose corn syrup, has been linked to a wide range of adverse health outcomes, including obesity, type 2 diabetes, cardiovascular disease, and various cancers (35). Similarly, artificial sweeteners (e.g., aspartame, saccharin, sucralose), which are commonly used in low-calorie products including tea, have also raised concerns about their potential metabolic and health impacts (36, 37). Previous studies have shown that high sugar intake is linked to an increased risk of chronic diseases, including cardiovascular diseases (38, 39). The consumption of added sugars can have adverse effects on blood lipid levels, resulting in insulin resistance, elevated levels of low-density lipoprotein cholesterol (LDL-C) and triglycerides, and reduced levels of high-density lipoprotein cholesterol (HDL-C). These factors are crucial contributors to metabolic syndrome and cardiovascular diseases, ultimately heightening the risk of heart disease and stroke (40). Furthermore, studies have shown that high sugar intake can increase the risk of various cancers, including breast cancer, colorectal cancer, and pancreatic cancer (41–43). It has been confirmed that the potential mechanisms behind this relationship involve insulin resistance, inflammation, and stimulation of the insulin-like growth factor 1 (IGF-1) pathway, thereby promoting tumor growth (44, 45). Similarly, studies have shown that artificial sweeteners may affect gut microbiota composition, potentially disrupting metabolism and immune responses. Such microbial imbalances have been linked to oxidative stress, DNA damage, and impaired cell signaling, thereby increasing the risk of chronic diseases, including CVD and cancer (46, 47). Although the evidence is mixed, in 2023, the World Health Organization (WHO) classified aspartame as a possible carcinogen in humans (12). These findings underscore the importance of limiting or avoiding the addition of sugar or artificial sweeteners to tea, as such additives may negate tea’s protective effects. Sugar addition contributes to excess caloric intake, weight gain, and obesity, which are well-established risk factors for CVD, type 2 diabetes, and several types of cancer. In our study, we also observed that individuals who used artificial sweeteners in tea tended to have a higher BMI. Habitual consumption of sweetened tea may contribute to adiposity and metabolic dysregulation, ultimately outweighing any potential health benefits of tea. Therefore, to maximize the potential health benefits of tea, it is advisable to consume tea without added sugars or sweeteners.

To the best of our knowledge, this study is the first to investigate the associations of tea consumption with all-cause, CVD, and cancer mortality, particularly focusing on unsweetened, sugar-sweetened, and artificially sweetened tea, in a prospective cohort study with large sample size and long enough follow-up. This study considers various confounding factors such as socioeconomic status, behavioral factors, and health conditions, and includes numerous sensitivity analyses to support the robustness of the results.

We also acknowledge the limitations of the present study. Firstly, this study is based on a specific population cohort (UK Biobank) and may not be representative of other populations or ethnic groups (48). However, this limitation does not affect the exploration of the relationship between exposure and outcome. Secondly, despite adjustments for various confounding factors, there may still be unmeasured or residual confounding that could influence the observed associations. Thirdly, this study relies on self-reported data for tea consumption, which may introduce recall bias and measurement error (49). These participants may have difficulty accurately recalling and reporting their tea consumption, leading to misclassificfation. Measurement error is unlikely to ever be entirely eliminated from dietary assessment, despite ongoing efforts to refine dietary assessment methods and reduce errors (such as multiple 24-h dietary recalls). Fourthly, the non-significant association between artificially sweetened tea consumption and mortality risk may reflect countervailing effects of sweeteners, residual confounding, or limited statistical power due to small subgroup sizes and few outcome events; therefore, these findings should be interpreted with caution. Finally, individuals with inconsistent tea consumption patterns across dietary recalls (overlapping consumers) were excluded to reduce exposure misclassification. However, this group may represent a behaviorally distinct population, and excluding them without separate analysis may limit the generalizability of our findings.

In summary, moderate consumption of unsweetened (3.5–4.5 drinks/day, with one drink defined as 250 mL or approximately 8.5 ounces) is significantly associated with a lower risk of all-cause mortality. However, sugar-sweetened and artificially sweetened tea could attenuate the protective effect of tea on cause-specific mortality. Our findings suggest that individuals who drink tea may consider moderating sugar and avoiding artificial sweeteners to potentially maximize health benefits.

## Data Availability

The datasets presented in this study can be found in online repositories. The names of the repository/repositories and accession number(s) can be found in the article/Supplementary material.
