# Supplementary material for: The associations of unsweetened, sugar-sweetened, and artificially sweetened tea consumption with all-cause and cause-specific mortality in 195,361 UK Biobank participants: a large prospective cohort study
Source: Front Nutr. 2025 Jul 31;12:1649279. doi: 10.3389/fnut.2025.1649279 (PMC12350117; doi:10.3389/fnut.2025.1649279)
Supplement: Supplementary file 1 [file Image_1.pdf]

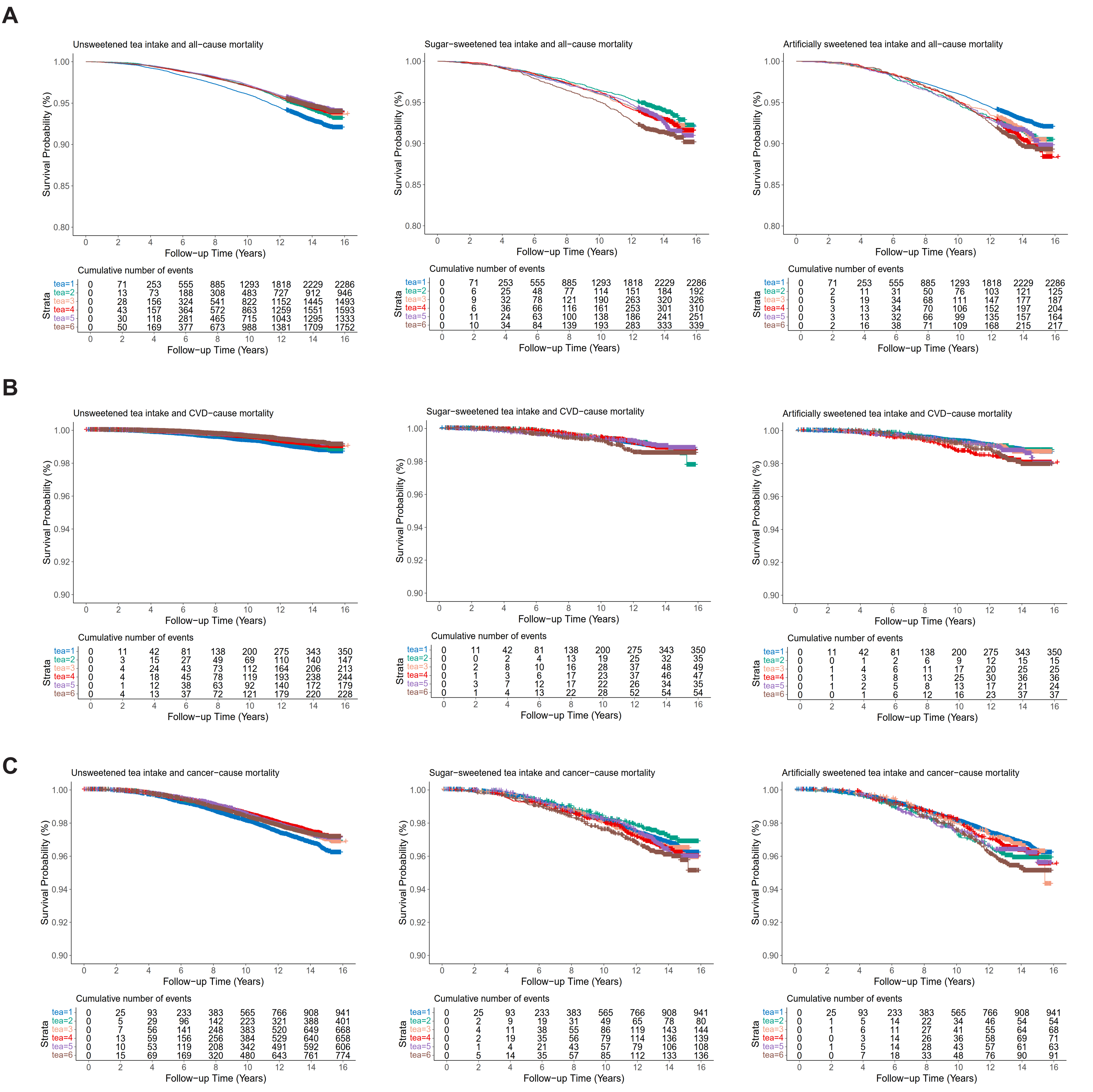

Supplementary Fig. S1 Kaplan–Meier survival curves illustrating the association between tea consumption and the cumulative mortality risk of (A) all-cause, (B) CVD, and (C) cancer.

Note: CVD, cardiovascular disease. Tea consumption categories are coded as follows: 1 = non-consumers; 2 = 0–1.5 drinks/day; 3 = 1.5–2.5 drinks/day; 4 = 2.5–3.5 drinks/day; 5 = 3.5–4.5 drinks/day; 6 = >4.5 drinks/day.

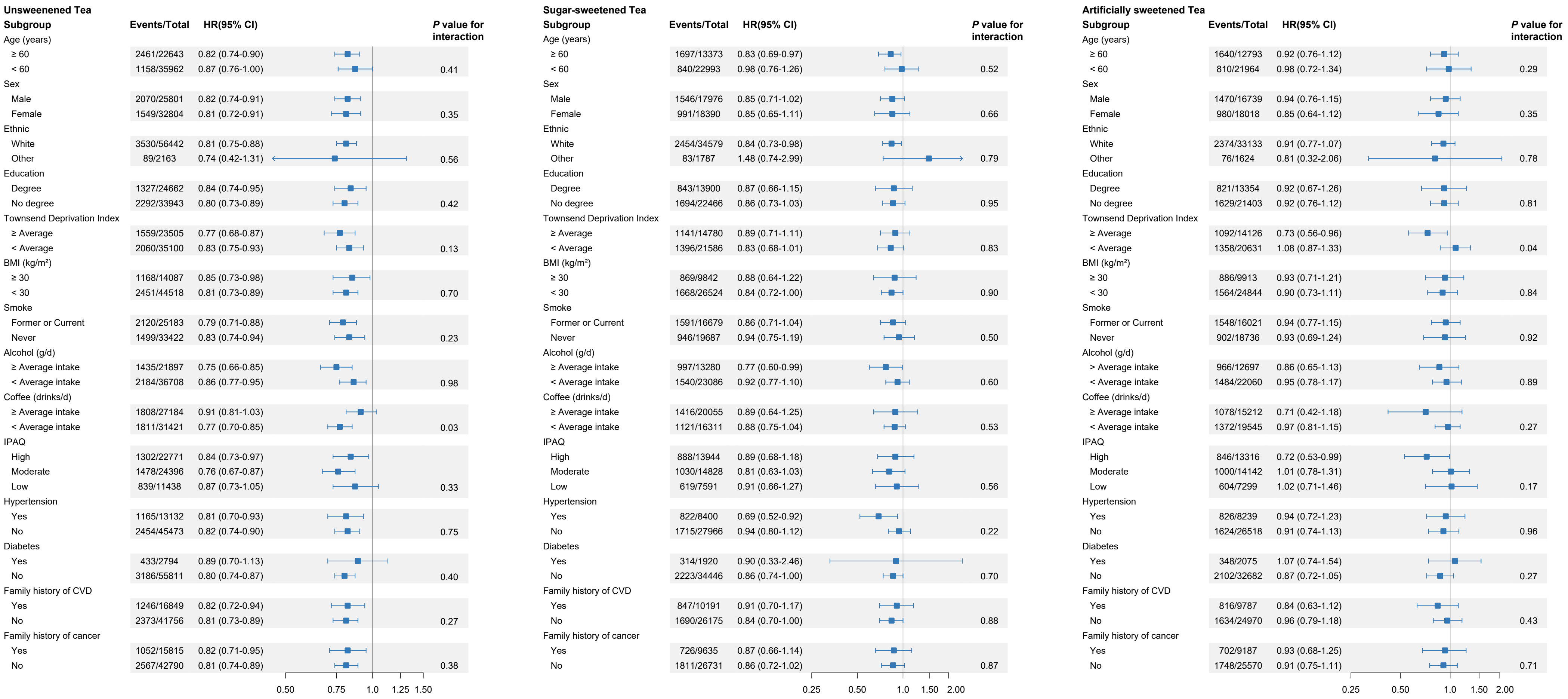

Supplementary Fig. S2 Association of unsweetened, sugar-sweetened, and artificially sweetened tea consumption and risk of all-cause mortality stratified by potential risk factors. Estimates are hazard ratios (95% CIs) from multivariable Cox proportional hazard models adjusted for age (continuous), gender, Townsend deprivation index (continuous), education level (degree or no degree), ethnicity (white or other), smoking status (current, former, or never), pack-years of smoking (continuous), Overall health (poor, fair, good, or excellent), Basal metabolic rate (continuous), physical activity level (low, moderate, or high), body mass index (continuous), hypertension (yes or no), diabetes (yes or no), depression (yes or no), family history of CVD disease (yes or no), family history of cancer (yes or no), long-standing illness (yes or no), cholesterol-lowering drug use (yes or no), blood pressure drug use (yes or no), vitamin and mineral supplement (yes or no), and intake of energy, total sugar, fresh fruit, vegetables, red meat, processed meat, alcohol, coffee, milk, naturally sweet juices, sugar-sweetened beverages, and artificially sweetened beverages.

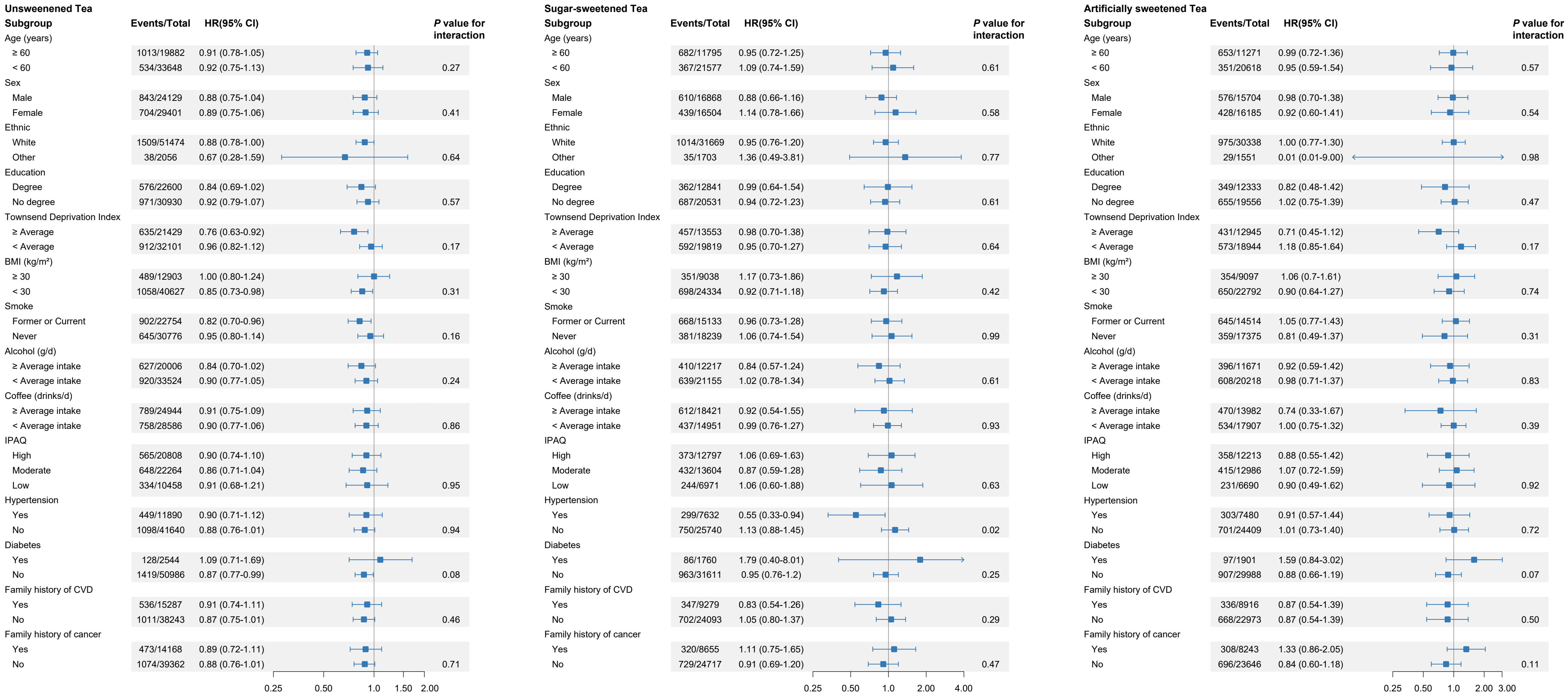

Unsweenened Tea

Subgroup	Events/Total	HR(95% CI)	*P* value for interaction
Age (years)			
≥ 60	1013/19882	0.91 (0.78-1.05)	
< 60	534/33648	0.92 (0.75-1.13)	0.27
Sex			
Male	843/24129	0.88 (0.75-1.04)	
Female	704/29401	0.89 (0.75-1.06)	0.41
Ethnic			
White	1509/51474	0.88 (0.78-1.00)	
Other	38/2056	0.67 (0.28-1.59)	0.64
Education			
Degree	576/22600	0.84 (0.69-1.02)	
No degree	971/30930	0.92 (0.79-1.07)	0.57
Townsend Deprivation Index			
≥ Average	635/21429	0.76 (0.63-0.92)	
< Average	912/32101	0.96 (0.82-1.12)	0.17
BMI (kg/m²)			
≥ 30	489/12903	1.00 (0.80-1.24)	
< 30	1058/40627	0.85 (0.73-0.98)	0.31
Smoke			
Former or Current	902/22754	0.82 (0.70-0.96)	
Never	645/30776	0.95 (0.80-1.14)	0.16
Alcohol (g/d)			
≥ Average intake	627/20006	0.84 (0.70-1.02)	
< Average intake	920/33524	0.90 (0.77-1.05)	0.24
Coffee (drinks/d)			
≥ Average intake	789/24944	0.91 (0.75-1.09)	
< Average intake	758/28586	0.90 (0.77-1.06)	0.86
IPAQ			
High	565/20808	0.90 (0.74-1.10)	
Moderate	648/22264	0.86 (0.71-1.04)	
Low	334/10458	0.91 (0.68-1.21)	0.95
Hypertension			
Yes	449/11890	0.90 (0.71-1.12)	
No	1098/41640	0.88 (0.76-1.01)	0.94
Diabetes			
Yes	128/2544	1.09 (0.71-1.69)	
No	1419/50986	0.87 (0.77-0.99)	0.08
Family history of CVD			
Yes	536/15287	0.91 (0.74-1.11)	
No	1011/38243	0.87 (0.75-1.01)	0.46
Family history of cancer			
Yes	473/14168	0.89 (0.72-1.11)	
No	1074/39362	0.88 (0.76-1.01)	0.71

Axis: 0.25 0.50 1.0 1.50 2.00

Sugar-sweetened Tea

Subgroup	Events/Total	HR(95% CI)	*P* value for interaction
Age (years)			
≥ 60	682/11795	0.95 (0.72-1.25)	
< 60	367/21577	1.09 (0.74-1.59)	0.61
Sex			
Male	610/16868	0.88 (0.66-1.16)	
Female	439/16504	1.14 (0.78-1.66)	0.58
Ethnic			
White	1014/31669	0.95 (0.76-1.20)	
Other	35/1703	1.36 (0.49-3.81)	0.77
Education			
Degree	362/12841	0.99 (0.64-1.54)	
No degree	687/20531	0.94 (0.72-1.23)	0.61
Townsend Deprivation Index			
≥ Average	457/13553	0.98 (0.70-1.38)	
< Average	592/19819	0.95 (0.70-1.27)	0.64
BMI (kg/m²)			
≥ 30	351/9038	1.17 (0.73-1.86)	
< 30	698/24334	0.92 (0.71-1.18)	0.42
Smoke			
Former or Current	668/15133	0.96 (0.73-1.28)	
Never	381/18239	1.06 (0.74-1.54)	0.99
Alcohol (g/d)			
≥ Average intake	410/12217	0.84 (0.57-1.24)	
< Average intake	639/21155	1.02 (0.78-1.34)	0.61
Coffee (drinks/d)			
≥ Average intake	612/18421	0.92 (0.54-1.55)	
< Average intake	437/14951	0.99 (0.76-1.27)	0.93
IPAQ			
High	373/12797	1.06 (0.69-1.63)	
Moderate	432/13604	0.87 (0.59-1.28)	
Low	244/6971	1.06 (0.60-1.88)	0.63
Hypertension			
Yes	299/7632	0.55 (0.33-0.94)	
No	750/25740	1.13 (0.88-1.45)	0.02
Diabetes			
Yes	86/1760	1.79 (0.40-8.01)	
No	963/31611	0.95 (0.76-1.2)	0.25
Family history of CVD			
Yes	347/9279	0.83 (0.54-1.26)	
No	702/24093	1.05 (0.80-1.37)	0.29
Family history of cancer			
Yes	320/8655	1.11 (0.75-1.65)	
No	729/24717	0.91 (0.69-1.20)	0.47

Axis: 0.25 0.50 1.0 2.00 4.00

Artificially sweetened Tea

Subgroup	Events/Total	HR(95% CI)	*P* value for interaction
Age (years)			
≥ 60	653/11271	0.99 (0.72-1.36)	
< 60	351/20618	0.95 (0.59-1.54)	0.57
Sex			
Male	576/15704	0.98 (0.70-1.38)	
Female	428/16185	0.92 (0.60-1.41)	0.54
Ethnic			
White	975/30338	1.00 (0.77-1.30)	
Other	29/1551	0.01 (0.01-9.00)	0.98
Education			
Degree	349/12333	0.82 (0.48-1.42)	
No degree	655/19556	1.02 (0.75-1.39)	0.47
Townsend Deprivation Index			
≥ Average	431/12945	0.71 (0.45-1.12)	
< Average	573/18944	1.18 (0.85-1.64)	0.17
BMI (kg/m²)			
≥ 30	354/9097	1.06 (0.7-1.61)	
< 30	650/22792	0.90 (0.64-1.27)	0.74
Smoke			
Former or Current	645/14514	1.05 (0.77-1.43)	
Never	359/17375	0.81 (0.49-1.37)	0.31
Alcohol (g/d)			
≥ Average intake	396/11671	0.92 (0.59-1.42)	
< Average intake	608/20218	0.98 (0.71-1.37)	0.83
Coffee (drinks/d)			
≥ Average intake	470/13982	0.74 (0.33-1.67)	
< Average intake	534/17907	1.00 (0.75-1.32)	0.39
IPAQ			
High	358/12213	0.88 (0.55-1.42)	
Moderate	415/12986	1.07 (0.72-1.59)	
Low	231/6690	0.90 (0.49-1.62)	0.92
Hypertension			
Yes	303/7480	0.91 (0.57-1.44)	
No	701/24409	1.01 (0.73-1.40)	0.72
Diabetes			
Yes	97/1901	1.59 (0.84-3.02)	
No	907/29988	0.88 (0.66-1.19)	0.07
Family history of CVD			
Yes	336/8916	0.87 (0.54-1.39)	
No	668/22973	0.87 (0.54-1.39)	0.50
Family history of cancer			
Yes	308/8243	1.33 (0.86-2.05)	
No	696/23646	0.84 (0.60-1.18)	0.11

Axis: 0.25 0.50 1.0 2.00 3.00

Supplementary Fig. S3 Association of unsweetened, sugar-sweetened, and artificially sweetened tea consumption and risk of cancer-cause mortality stratified by potential risk factors. Estimates are hazard ratios (95% CIs) from multivariable Cox proportional hazard models adjusted for age (continuous), gender, Townsend deprivation index (continuous), education level (degree or no degree), ethnicity (white or other), smoking status (current, former, or never), pack-years of smoking (continuous), Overall health (poor, fair, good, or excellent), Basal metabolic rate (continuous), physical activity level (low, moderate, or high), body mass index (continuous), hypertension (yes or no), diabetes (yes or no), depression (yes or no), family history of CVD disease (yes or no), family history of cancer (yes or no), long-standing illness (yes or no), cholesterol-lowering drug use (yes or no), blood pressure drug use (yes or no), vitamin and mineral supplement (yes or no), and intake of energy, total sugar, fresh fruit, vegetables, red meat, processed meat, alcohol, coffee, milk, naturally sweet juices, sugar-sweetened beverages, and artificially sweetened beverages.

Unsweenened Tea

Subgroup	Events/Total	HR(95% CI)	*P* value for interaction
Age (years)			
≥ 60	385/20922	0.80 (0.63-1.02)	
< 60	144/35138	0.71 (0.46-1.10)	0.19
Sex			
Male	351/24014	0.75 (0.58-0.97)	
Female	178/32046	0.81 (0.57-1.16)	0.94
Ethnic			
White	512/53974	0.75 (0.61-0.93)	
Other	17/2086	1.36 (0.36-5.19)	0.84
Education			
Degree	221/23924	0.87 (0.63-1.20)	
No degree	208/32136	0.72 (0.54-0.95)	0.67
Townsend Deprivation Index			
≥ Average	226/22458	0.61 (0.43-0.86)	
< Average	303/33602	0.87 (0.66-1.14)	0.07
BMI (kg/m²)			
≥ 30	184/13128	0.66 (0.45-0.96)	
< 30	345/42932	0.85 (0.66-1.09)	0.62
Smoke			
Former or Current	295/23655	0.76 (0.57-1.02)	
Never	234/32405	0.76 (0.56-1.04)	0.81
Alcohol (g/d)			
≥ Average intake	212/20941	0.64 (0.46-0.91)	
< Average intake	317/35119	0.87 (0.67-1.14)	0.48
Coffee (drinks/d)			
≥ Average intake	264/25905	0.82 (0.59-1.15)	
< Average intake	265/30155	0.71 (0.55-0.92)	0.45
IPAQ			
High	206/21861	0.86 (0.62-1.21)	
Moderate	201/23378	0.74 (0.50-1.09)	
Low	122/10821	0.64 (0.38-1.07)	0.18
Hypertension			
Yes	261/13047	0.89 (0.66-1.20)	
No	268/43013	0.69 (0.52-0.93)	0.24
Diabetes			
Yes	77/2367	0.64 (0.35-1.17)	
No	452/53693	0.79 (0.63-0.99)	0.66
Family history of CVD			
Yes	195/15874	0.64 (0.45-0.91)	
No	334/40186	0.85 (0.66-1.11)	0.61
Family history of cancer			
Yes	132/15147	0.91 (0.60-1.38)	
No	397/40913	0.72 (0.56-0.92)	0.38

Axis: 0.50, 1.0, 2.00

Sugar-sweetened Tea

Subgroup	Events/Total	HR(95% CI)	*P* value for interaction
Age (years)			
≥ 60	268/12177	0.86 (0.55-1.35)	
< 60	117/22394	0.85 (0.39-1.84)	0.65
Sex			
Male	267/16679	0.85 (0.54-1.32)	
Female	118/17892	0.86 (0.40-1.85)	0.83
Ethnic			
White	369/32848	0.81 (0.54-1.21)	
Other	16/1723	2.82 (0.55-14.5)	0.57
Education			
Degree	151/13432	1.18 (0.63-2.22)	
No degree	234/21139	0.69 (0.42-1.14)	0.41
Townsend Deprivation Index			
≥ Average	172/14038	0.73 (0.40-1.34)	
< Average	213/20533	0.92 (0.55-1.53)	0.77
BMI (kg/m²)			
≥ 30	146/9148	1.15 (0.55-2.40)	
< 30	239/25423	0.75 (0.48-1.19)	0.31
Smoke			
Former or Current	227/15535	0.90 (0.55-1.47)	
Never	158/19036	0.84 (0.44-1.58)	0.42
Alcohol (g/d)			
≥ Average intake	158/12621	0.79 (0.43-1.44)	
< Average intake	227/21950	0.91 (0.55-1.50)	0.55
Coffee (drinks/d)			
≥ Average intake	214/19025	1.10 (0.48-2.53)	
< Average intake	171/15546	0.83 (0.53-1.29)	0.44
IPAQ			
High	142/13326	1.19 (0.67-2.11)	
Moderate	147/14124	0.84 (0.40-1.77)	
Low	96/7121	0.38 (0.14-1.05)	0.12
Hypertension			
Yes	186/8349	0.82 (0.45-1.49)	
No	199/26222	0.92 (0.56-1.53)	0.99
Diabetes			
Yes	59/1616	1.56 (0.01-9.00)	
No	326/32955	0.86 (0.58-1.27)	0.99
Family history of CVD			
Yes	145/9501	1.02 (0.56-1.88)	
No	240/25070	0.74 (0.45-1.24)	0.40
Family history of cancer			
Yes	93/9167	1.08 (0.51-2.28)	
No	292/25404	0.76 (0.48-1.21)	0.51

Axis: 0.25, 0.50, 1.0, 2.00, 3.00

Artificially sweetened Tea

Subgroup	Events/Total	HR(95% CI)	*P* value for interaction
Age (years)			
≥ 60	262/11638	1.01 (0.63-1.61)	
< 60	112/21380	0.56 (0.17-1.81)	0.30
Sex			
Male	257/15484	0.92 (0.55-1.54)	
Female	117/17534	0.80 (0.36-1.79)	0.92
Ethnic			
White	357/31457	0.77 (0.48-1.23)	
Other	17/1561	6.88 (1.94-24.50)	0.01
Education			
Degree	144/12897	0.74 (0.32-1.72)	
No degree	230/20121	0.95 (0.57-1.58)	0.54
Townsend Deprivation Index			
≥ Average	166/13397	0.65 (0.31-1.35)	
< Average	208/19621	1.17 (0.68-2.00)	0.26
BMI (kg/m²)			
≥ 30	146/9210	0.77 (0.38-1.55)	
< 30	228/23808	1.00 (0.57-1.72)	0.65
Smoke			
Former or Current	218/14911	0.79 (0.45-1.40)	
Never	156/18107	1.20 (0.61-2.34)	0.41
Alcohol (g/d)			
≥ Average intake	154/12059	1.00 (0.52-1.90)	
< Average intake	220/20959	0.84 (0.47-1.51)	0.31
Coffee (drinks/d)			
≥ Average intake	171/14432	0.76 (0.24-2.43)	
< Average intake	203/18586	0.99 (0.62-1.57)	0.70
IPAQ			
High	128/12703	0.65 (0.28-1.51)	
Moderate	151/13484	1.51 (0.84-2.17)	
Low	95/6831	0.46 (0.11-1.94)	0.24
Hypertension			
Yes	191/8189	1.38 (0.83-2.28)	
No	183/24829	0.44 (0.18-1.08)	0.04
Diabetes			
Yes	67/1747	1.37 (0.61-3.09)	
No	307/31271	0.79 (0.47-1.32)	0.35
Family history of CVD			
Yes	141/9129	1.01 (0.53-1.92)	
No	233/23889	0.84 (0.46-1.51)	0.50
Family history of cancer			
Yes	89/8742	0.79 (0.31-2.00)	
No	285/24276	0.94 (0.58-1.54)	0.98

Axis: 0.25, 0.50, 1.0, 2.00, 4.00

Supplementary Fig. S4 Association of unsweetened, sugar-sweetened, and artificially sweetened tea consumption and risk of CVD-cause mortality stratified by potential risk factors. CVD, cardiovascular disease. Estimates are hazard ratios (95% CIs) from multivariable Cox proportional hazard models adjusted for age (continuous), gender, Townsend deprivation index (continuous), education level (degree or no degree), ethnicity (white or other), smoking status (current, former, or never), pack-years of smoking (continuous), Overall health (poor, fair, good, or excellent), Basal metabolic rate (continuous), physical activity level (low, moderate, or high), body mass index (continuous), hypertension (yes or no), diabetes (yes or no), depression (yes or no), family history of CVD disease (yes or no), family history of cancer (yes or no), long-standing illness (yes or no), cholesterol-lowering drug use (yes or no), blood pressure drug use (yes or no), vitamin and mineral supplement (yes or no), and intake of energy, total sugar, fresh fruit, vegetables, red meat, processed meat, alcohol, coffee, milk, naturally sweet juices, sugar-sweetened beverages, and artificially sweetened beverages.
