# Supplementary material for: The associations of unsweetened, sugar-sweetened, and artificially sweetened tea consumption with all-cause and cause-specific mortality in 195,361 UK Biobank participants: a large prospective cohort study
Source: Front Nutr. 2025 Jul 31;12:1649279. doi: 10.3389/fnut.2025.1649279 (PMC12350117; doi:10.3389/fnut.2025.1649279)
Supplement: Supplementary file 2 [file Table_1.pdf]

Table S1. Classification of tea consumers by sweeteners.

Categories of tea consumers	N	%
**Non-consumers**	33395	16.2%
**Sole-consumers**		
Unsweetened	136180	66.1%
Sugar-sweetened	20559	10.0%
Artificially sweetened	10522	5.1%
**Overlapping-consumers of sweeteners**		
Unsweetened & Sugar-sweetened	2139	1.0%
Unsweetened & Artificially sweetened	750	0.4%
Sugar-sweetened & Artificially sweetened	2364	1.1%
Unsweetened & Sugar-sweetened & Artificially sweetened	89	0.0%
**Total**	205998	100%

Note: As shown in the workflow, a total of 210,950 individuals completed at least one 24-hour dietary recall questionnaire, and 205,998 individuals remained after excluding pregnancy, extreme total energy intake, loss of follow-up, and no tea drinking information. Then, we excluded the overlapping consumption of unsweetened, sweetened and artificially sweetened tea (n =5342) to reduce misclassification.

Table S2. Detailed information on missing covariates.

Covariates	N*	Missing rate (%)
Ethnicity	567	0.003
Townsend index	231	0.001
Education	751	0.004
Smoking status	372	0.002
Pack-years of smoking	28822	0.148
Overall health	366	0.002
Basal metabolic rate	2640	0.014
IPAQ	28444	0.146
BMI	469	0.002
Tea on average in past year	136	0.001
Hypertension	191	0.001
Diabetes	264	0.001
Depression	2635	0.013
Self-reported cancer	256	0.001
Self-reported CVD	194	0.001
Family history of CVD	2968	0.015
Family history of cancer	2968	0.015
Long-standing illness	3634	0.019
Cholesterol-lowering drug	635	0.003
Blood pressure drug	635	0.003
Vitamin/mineral use	402	0.002
ABSs	4398	0.023
SSBs	4398	0.023
NSJs	4398	0.023
Coffee	163	0.001

Abbreviations: IPAQ, International Physical Activity Questionnaire; BMI, body mass index (calculated as weight in kilograms divided by height in meters squared); CVD, cardiovascular disease; ABS, artificially sweetened beverage; SSB, sugar-sweetened beverage; NSJ, naturally sweet juices.

*N represents the number of missing responses

Table S3. Mean (mean ±SD) consumption of sugar and artificially sweetener added to tea across multiple 24-hour dietary recalls.

The number of times participants completed 24-hour dietary recalls	Number of participants (%)		Mean consumption (mean ± SD)	
	Sugar-sweetened consumers	Artificially sweetened consumers	Sugar added to tea (teaspoons/drink)	Artificially sweetener added to tea (teaspoons/drink)
**1 time**	9163 (46.4%)	4596 (45.5%)	1.1 ± 0.6	1.4 ± 0.6
**2 times**	4351 (22.0%)	2340 (23.2%)	1.1 ± 0.6	1.4 ± 0.6
**3 times**	3444 (17.4%)	1768 (17.5%)	1.1 ± 0.5	1.4 ± 0.6
**4 times**	2378 (12.0%)	1208 (12.0%)	1.1 ± 0.5	1.4 ± 0.6
**5 times**	416 (2.1%)	194 (1.9%)	1.0 ± 0.5	1.4 ± 0.5
**Total**	19752 (100.0%)	10106 (100.0%)	1.1 ± 0.6	1.4 ± 0.6

Table S4. Spearman correlation coefficients of tea consumption across multiple 24-hour dietary recalls and baseline questionnaire.

The number of times participants completed 24-hour dietary recalls	Number of tea consumers (%)	Consumption of tea (drink/d) in each dietary recall (mean ± SD)		Spearman correlation coefficients across 24-hour dietary recalls and tea intake at baseline					
				baseline	1st	2nd	3rd	4th	5th
**1 time**	69959	3.1 ± 1.6	1st	0.70	-				
**2 times**	38228	3.2 ± 1.6	1st	0.67	-	0.70			
		3.2 ± 1.5	2nd	0.63	-	-			
**3 times**	30826	3.3 ± 1.6	1st	0.67	-	0.70	0.69		
		3.3 ± 1.5	2nd	0.63	-	-	0.70		
		3.3 ± 1.5	3rd	0.62	-	-	-		
**4 times**	19945	3.5 ± 1.5	1st	0.67	-	0.69	0.70	0.68	
		3.4 ± 1.5	2nd	0.62	-	-	0.70	0.69	
		3.4 ± 1.5	3rd	0.62	-	-	-	0.70	
		3.4 ± 1.5	4th	0.60	-	-	-	-	
**5 times**	3558	3.5 ± 1.5	1st	0.77	-	0.69	0.68	0.68	0.66
		3.5 ± 1.5	2nd	0.68	-	-	0.69	0.72	0.70
		3.4 ± 1.5	3rd	0.66	-	-	-	0.71	0.70
		3.5 ± 1.5	4th	0.66	-	-	-	-	0.71
		3.5 ± 1.5	5th	0.65	-	-	-	-	-
**Total**	162516								

Table S5. Combined Tea Consumption Summary by Type and Sweetener Category in the UK Biobank Cohort.

Tea Type	Sweetener Type	Non-drinkers	Drinkers	Total
All Tea Types	Non-consumers	32845	0	32845
	unsweetened	0	132658	132658
	Sugar-sweetened	0	19752	19752
	Artificially sweetened	0	10106	10106
Standard Tea	Non-consumers	32845	0	32845
	unsweetened	15762	116896	132658
	Sugar-sweetened	763	18989	19752
	Artificially sweetened	652	9454	10106
rooibos/redbush tea	Non-consumers	32845	0	32845
	unsweetened	123286	9372	132658
	Sugar-sweetened	19250	502	19752
	Artificially sweetened	9790	316	10106
green tea	Non-consumers	32845	0	32845
	unsweetened	116787	15871	132658
	Sugar-sweetened	18986	766	19752
	Artificially sweetened	9655	451	10106
herbal or fruit tea	Non-consumers	32845	0	32845
	unsweetened	107688	24970	132658
	Sugar-sweetened	18441	1311	19752
	Artificially sweetened	9315	791	10106
other tea or infusion	Non-consumers	32845	0	32845
	unsweetened	123836	8822	132658
	Sugar-sweetened	19068	684	19752
	Artificially sweetened	9663	443	10106

Table S6. Components and scoring of the Alternative Healthy Eating Index (AHEI)-2010 adherence score.

Diet component	UKB variable	label	Coding (standard servings per day)	Scoring	
				Criteria for minimum score(0)	Criteria for maximum score (10)
Vegetable	103990	Vegetable consumers	0/1, N/Y		
	104060	Mixed vegetable	444/555/1/2/300, ¼ / ½ /1/2/3+		
	104070	Vegetable pieces	444/555/1/2/300, ¼ / ½ /1/2/3+		
	104080	Coleslaw	444/555/1/2/300, ¼ / ½ /1/2/3+		
	104090	Side salad	444/555/1/2/300, ¼ / ½ /1/2/3+		
	104100	Avocado	444/555/1/2/300, ¼ / ½ /1/2/3+		
	104120	Green bean	444/555/1/2/300, ¼ / ½ /1/2/3+		
	104130	Beetroot	444/555/1/2/300, ¼ / ½ /1/2/3+		
	104140	Broccoli	444/555/1/2/300, ¼ / ½ /1/2/3+		
	104150	Butternut squash	444/555/1/2/300, ¼ / ½ /1/2/3+		
	104160	Cabbage/kale	444/555/1/2/300, ¼ / ½ /1/2/3+		
	104170	Carrot	444/555/1/2/300, ¼ / ½ /1/2/3+		
	104180	Cauliflower	444/555/1/2/300, ¼ / ½ /1/2/3+		
	104190	Celery	444/555/1/2/300, ¼ / ½ /1/2/3+		
	104200	Courgette	444/555/1/2/300, ¼ / ½ /1/2/3+		
	104210	Cucumber	444/555/1/2/300, ¼ / ½ /1/2/3+		
	104220	Garlic	444/555/1/2/300, ¼ / ½ /1/2/3+		
	104230	Leek	444/555/1/2/300, ¼ / ½ /1/2/3+		
	104240	Lettuce	444/555/1/2/300, ¼ / ½ /1/2/3+		
	104250	Mushroom	444/555/1/2/300, ¼ / ½ /1/2/3+		
	104260	Onion	444/555/1/2/300, ¼ / ½ /1/2/3+		
	104270	Parsnip	444/555/1/2/300, ¼ / ½ /1/2/3+		
	104280	Pea	444/555/1/2/300, ¼ / ½ /1/2/3+		
	104290	Sweet pepper	444/555/1/2/300, ¼ / ½ /1/2/3+		
	104300	Spinach	444/555/1/2/300, ¼ / ½ /1/2/3+		
	104310	Sprouts	444/555/1/2/300, ¼ / ½ /1/2/3+		
	104320	Sweetcorn	444/555/1/2/300, ¼ / ½ /1/2/3+		
	104330	Sweet potato	444/555/1/2/300, ¼ / ½ /1/2/3+		

	104340	Fresh tomato	444/555/1/2/300, ¼ / ½ /1/2/3+		
	104350	Tinned tomato	444/555/1/2/300, ¼ / ½ /1/2/3+		
	104360	Turnip/swede	444/555/1/2/300, ¼ / ½ /1/2/3+		
	104370	Watercress	444/555/1/2/300, ¼ / ½ /1/2/3+		
	104380	Other vegetables	444/555/1/2/300, ¼ / ½ /1/2/3+		
	102520	Soup consumers	0/1, N/Y		
	102540	Canned soup intake *	555/1/2/3/400, ½ /1/2/3/4+		
	102620	Homemade soup intake *	555/1/2/3/400, ½ /1/2/3/4+		
	20108	Ingredients in canned soup	5, vegetables		
	20109	Ingredients in homemade soup	5, vegetables		
	103310	Spreads/sauces consumers	0/1, N/Y		
	20088	Types of spreads/sauces consumed			
		Tomato sauce	346 (max 1 serving)		
	*1veg soup= 0.25 veg serving<br>Since 20108, and 20109 are not exclusive, we derived a "servings of vegetable soup" variable weighted by response.<br>Since 20088 is not exclusive, we derived a "servings of vegetable sauce" variable weighted by response (assumed max 1 serving).<br>Used mean intake across diet records for each item then summed item means for daily servings.			0 serving/day	≥5 serving/day
Fruit (not juice)	104400	Fruit consumers	0/1, N/Y		
	104410	Stewed fruit	555/1/2/3/400, ½ /1/2/3/4+		
	104420	Prune	555/1/2/3/400, ½ /1/2/3/4+		
	104430	Dried fruit	555/1/2/3/400, ½ /1/2/3/4+		
	104440	Mixed fruit	555/1/2/3/400, ½ /1/2/3/4+		
	104450	Apple	555/1/2/3/400, ½ /1/2/3/4+		

	104460	Banana	555/1/2/3/400, ½ /1/2/3/4+		
	104470	Berry	555/1/2/3/400, ½ /1/2/3/4+		
	104480	Cherry	555/1/2/3/400, ½ /1/2/3/4+		
	104490	Grapefruit	555/1/2/3/400, ½ /1/2/3/4+		
	104500	Grape	555/1/2/3/400, ½ /1/2/3/4+		
	104510	Mango	555/1/2/3/400, ½ /1/2/3/4+		
	104520	Melon	555/1/2/3/400, ½ /1/2/3/4+		
	104530	Orange	555/1/2/3/400, ½ /1/2/3/4+		
	104540	Satsuma	555/1/2/3/400, ½ /1/2/3/4+		
	104550	Peach/nectarine	555/1/2/3/400, ½ /1/2/3/4+		
	104560	Pear	555/1/2/3/400, ½ /1/2/3/4+		
	104570	Pineapple	555/1/2/3/400, ½ /1/2/3/4+		
	104580	Plum	555/1/2/3/400, ½ /1/2/3/4+		
	104590	Other fruit	555/1/2/3/400, ½ /1/2/3/4+		
	Used mean intake across diet records for each item then summed item means for daily servings.			0 serving/day	≥4 serving/day
Whole grains	100760	Breakfast cereal consumed	0/1, N/Y		
	100770	Porridge	555/1/200, ½ /1/2+		
	100800	Muesli *	555/1/200, ½ /1/2+		
	100810	Oat crunch	555/1/200, ½ /1/2+		
	100840	Bran cereal	555/1/200, ½ /1/2+		
	100850	Whole-wheat cereal	555/1/200, ½ /1/2+		
	100940	Bread consumed	0/1, N/Y		
	100950	Sliced bread intake	555/1/2/3/4/5/600, ½ /1/2/3/4/5/6+		
	20091	Type of sliced bread eaten	1/2/3/4/5, whi/mix/ww/seed/oth		
	101020	Baguette	555/1/2/3/4/5/600, ½ /1/2/3/4/5/6+		
	20092	Type of baguette eaten	1/2/3/4/5, whi/mix/ww/seed/oth		
	101090	Bap	555/1/2/3/4/5/600, ½ /1/2/3/4/5/6+		
	20093	Type of large bap eaten	1/2/3/4/5, whi/mix/ww/seed/oth		
	101160	Bread roll	555/1/2/3/4/5/600, ½ /1/2/3/4/5/6+		

	20094	Type of bread roll eaten	1/2/3/4/5, whi/mix/ww/seed/oth		
	101250	Crispbread	555/1/2/3/4/5/600, ½ /1/2/3/4/5/6+		
	101260	Oatcakes	555/1/2/3/4/5/600, ½ /1/2/3/4/5/6+		
	102700	Starchy food consumers	0/1, N/Y		
	102720	Wholemeal pasta	555/1/200, ½ /1/2+		
	102740	Brown rice	555/1/200, ½ /1/2+		
	102780	Other grain ("bulgar, wheat, millett, barley")	555/1/200, ½ /1/2+		
	*Assumed whole grains contributed half the weight of Muesli (fruit/nuts the other half). Since 20091, 20092, 20093, 20094 are not exclusive, we derived a "servings of whole-wheat bread" variable weighted by response. Used mean intake across diet records for each item then summed item means for daily servings.<br>One serving of a 100% whole-grain product (i.e., 0.5 cup of oatmeal or brown rice, or one slice of whole-grain bread) contains~15–20 g of whole grains (per dry weight). Used mean intake across diet records for each item then summed item means for daily servings.			0 serving/day	5 serving/day (Female)<br>6 serving/day (Male)
Sugar-sweetened beverages and fruit juice	100170	Fizzy drink	555/1/2/3/4/5/600, ½ /1/2/3/4/5/6+		
	100180	Squash	555/1/2/3/4/5/600, ½ /1/2/3/4/5/6+		
	100190	Orange juice	555/1/2/3/4/5/600, ½ /1/2/3/4/5/6+		
	100200	Grapefruit juice	555/1/2/3/4/5/600, ½ /1/2/3/4/5/6+		
	100210	Pure fruit/vegetable juice *	555/1/2/3/4/5/600, ½ /1/2/3/4/5/6+		
	100220	Fruit smoothie	555/1/2/3/4/5/600, ½ /1/2/3/4/5/6+		
	100510	Other non-alcoholic drinks	0/1, N/Y		
	100530	Flavoured milk	555/1/2/3/4/5/600, ½ /1/2/3/4/5/6+		
	100550	Hot chocolate	555/1/2/3/4/5/600, ½ /1/2/3/4/5/6+		
	* 1 serving fruit or vegetable juice = 1/2 fruit juice serving<br>Used mean intake across diet records for each item then summed item means for daily intake.			⩾1 serving/day	0 serving/day
Nut& Legumes	103990	Vegetable consumers	0/1, N/Y		
	104000	Baked bean	444/555/1/2/300, ¼ / ½ /1/2/3+		
	104010	Pulses	444/555/1/2/300, ¼ / ½ /1/2/3+		

	104110	Broad bean	444/555/1/2/300, ¼ / ½ /1/2/3+		
	102520	Soup consumer	0/1, N/Y		
	102540	Canned	555/1/2/3/400, ½ /1/2/3/4+		
	102620	Homemade	555/1/2/3/400, ½ /1/2/3/4+		
	20108	Ingredients in canned soup	4, pulses		
	20109	Ingredients in homemade soup<br>*1 bean soup= 0.25 bean serving	4, pulses		
	103310	Spreads/sauces consumers	0/1, N/Y		
	20088	Types of spreads/sauces consumed	Peanut butter/Hummus, 334/336		
	102400	Savoury snack consumers	0/1, N/Y		
	102410	Salted peanuts	555/1/2/300, ½ /1/2/3+		
	102420	Unsalted peanuts	555/1/2/300, ½ /1/2/3+		
	102430	Salted nuts	555/1/2/300, ½ /1/2/3+		
	102440	Unsalted nuts	555/1/2/300, ½ /1/2/3+		
	102450	Seeds	555/1/2/300, ½ /1/2/3+		
	103250	Vegetarian alternatives	0/1, N/Y		
	103270	Tofu	555/1/2/3/400, ½ /1/2/3/4+		
	Since 20108, and 20109 are not exclusive, we derived a "servings of legume soup" variable weighted by response.<br>Since 20088 is not exclusive, we derived a "servings of legume sauce" variable weighted by response (assumed max 1 serving).<br>Used mean intake across diet records for each item then summed item means for daily servings.			0 serving/day	≥1 serving/day
Red meat & products	103000	Meat consumers	0/1, N/Y		
	103010	Sausage	555/1/2/3/4/500, ½ /1/2/3/4/5+		
	103020	Beef	555/1/2/3/4/500, ½ /1/2/3/4/5+		
	103030	Pork	555/1/2/3/4/500, ½ /1/2/3/4/5+		

	103040	Lamb	555/1/2/3/4/500, ½ /1/2/3/4/5+		
	103070	Bacon	555/1/2/3/4/500, ½ /1/2/3/4/5+		
	103080	Ham	555/1/2/3/4/500, ½ /1/2/3/4/5+		
	Used mean intake across diet records for each item then summed item means for daily servings.			≥1.5 serving/day	0 serving/day
Trans fat	26155	Trans fatty acids	g/day		
	26002	Energy	KJ/day		
	Converted g to calories (1 g ≈ 9 kcal) and derived % of total energy (1 kcal≈4.184 kJ). Used mean intake across diet records.			≥4% energy	≤0.5% energy
Long chain (n-3) fats (EPA +DHA)	103140	Fish consumer	0/1, N/Y		
	103160	Oily fish	555/1/2/3/400, ½ /1/2/3/4+		
	The cutoff for optimal intake (250 mg/d) is ~2 4-oz servings of fish /week, which is consistent with current guidelines (1 oz = 28.35 g). We applied the cutoff for optimal intake of 2 servings per week.			0 serving/week	≥2 serving/week
Polyunsaturated fatty acids (PUFA), % energy	26015	n-3 fatty acids	g/day		
	26016	n-3 fatty acids	g/day		
	26002	Energy	KJ/day		
	Converted g to calories (1 g ≈9 kcal) and derived % of total energy (1 kcal≈4.184 kJ). Used mean intake across diet records.			≤2 % energy	≥10 % energy
Sodium	26052	Sodium	mg/day		
	Used mean intake across diet records.			Highest decile	Lowest decile
Alcohol	100580	Alcohol consumed	0/1, N/Y		
	100022	Alcohol	g/d		
	26030	Alcohol	g/d		
	One drink/day represents 4 oz of wine, 12 oz of beer, or 1.5 oz of spirits. Used mean intake across diet records.			≥2.5 drink/d (Female)<br>≥3.5 drink/d (Male)	0.5-1.5 drink/d (Female)<br>0.5-2.0 drink/d (Male)

Note: N: no; Y: yes.

Table S7. Associations of tea consumption with all-cause mortality stratified by genetic caffeine metabolism score (wCMS$_{G4}$) in individuals who had genetic data.

Outcome	Events/Model	Nonconsumers	Tea consumers				
			0-1.5 drinks/d	1.5-2.5 drinks/d	2.5-3.5 drinks/d	3.5-4.5 drinks/d	>4.5 drinks/d
**Unsweetened tea**							
0-2	Events, n (%)	217 (6.6)	92 (5.1)	143 (4.8)	179 (5.9)	133 (5.2)	138 (4.6)
	Multivariable model*	1 (Reference)	0.84 (0.65-1.08)	0.75 (0.60-0.93)	0.89 (0.72-1.10)	0.79 (0.62-1.00)	0.73 (0.58-0.93)
2-3	Events, n (%)	504 (6.6)	225 (5.6)	353 (5.4)	376 (5.4)	307 (5.2)	404 (5.6)
	Multivariable model*	1 (Reference)	0.95 (0.81-1.11)	0.89 (0.77-1.02)	0.89 (0.77-1.03)	0.86 (0.74-1.01)	0.91 (0.79-1.06)
3-4	Events, n (%)	661 (6.7)	253 (5.3)	443 (5.3)	469 (5.4)	368 (4.9)	661 (5.4)
	Multivariable model*	1 (Reference)	0.87 (0.75-1.01)	0.84 (0.74-0.95)	0.86 (0.76-0.98)	0.76 (0.66-0.87)	0.86 (0.75-0.98)
>4	Events, n (%)	842 (7.4)	356 (6.3)	517 (5.5)	531 (5.2)	485 (5.3)	656 (5.3)
	Multivariable model*	1 (Reference)	0.96 (0.84-1.09)	0.82 (0.73-0.92)	0.77 (0.69-0.87)	0.80 (0.70-0.90)	0.80 (0.71-0.90)
**Sugar-sweetened tea**							
0-2	Events, n (%)	217 (6.6)	23 (5.6)	35 (6.2)	32 (5.8)	23 (5.9)	29 (8.5)
	Multivariable model*	1 (Reference)	0.77 (0.49-1.20)	0.88 (0.60-1.29)	0.89 (0.59-1.33)	0.71 (0.45-1.14)	0.89 (0.57-1.40)
2-3	Events, n (%)	504 (6.6)	43 (5.1)	72 (6.3)	77 (7.0)	57 (6.9)	78 (9.1)
	Multivariable model*	1 (Reference)	0.77 (0.56-1.06)	0.95 (0.73-1.23)	1.02 (0.79-1.32)	0.90 (0.67-1.22)	0.94 (0.71-1.24)
3-4	Events, n (%)	661 (6.7)	58 (6.3)	112 (7.8)	79 (6.4)	81 (7.7)	97 (8.3)
	Multivariable model*	1 (Reference)	0.83 (0.63-1.10)	1.10 (0.89-1.36)	0.82 (0.64-1.06)	0.91 (0.71-1.18)	0.79 (0.61-1.02)
>4	Events, n (%)	842 (7.4)	58 (6.0)	104 (7.0)	120 (8.2)	84 (7.1)	134 (9.1)
	Multivariable model*	1 (Reference)	0.78 (0.59-1.02)	0.90 (0.73-1.11)	0.97 (0.79-1.19)	0.80 (0.62-1.02)	0.90 (0.73-1.13)
**Artificially sweetened tea**							
0-2	Events, n (%)	217 (6.6)	18 (11.0)	14 (5.6)	26 (11.1)	14 (8.2)	19 (10.7)
	Multivariable model*	1 (Reference)	1.05 (0.64-1.72)	0.65 (0.37-1.12)	1.12 (0.73-1.73)	0.77 (0.44-1.35)	1.04 (0.63-1.73)
2-3	Events, n (%)	504 (6.6)	25 (6.8)	61 (11.7)	49 (9.5)	40 (8.9)	48 (10.5)
	Multivariable model*	1 (Reference)	0.84 (0.56-1.25)	1.26 (0.96-1.65)	0.96 (0.70-1.30)	1.14 (0.81-1.59)	1.04 (0.76-1.43)
3-4	Events, n (%)	661 (6.7)	31 (7.5)	60 (9.0)	53 (7.9)	41 (7.5)	71 (10.0)
	Multivariable model*	1 (Reference)	0.83 (0.57-1.19)	1.02 (0.78-1.34)	0.82 (0.61-1.09)	0.83 (0.60-1.15)	0.92 (0.70-1.20)
>4	Events, n (%)	842 (7.4)	50 (9.8)	46 (6.2)	73 (9.7)	64 (9.1)	74 (8.8)
	Multivariable model*	1 (Reference)	1.04 (0.78-1.38)	0.62 (0.46-0.84)	0.96 (0.75-1.23)	0.85 (0.65-1.10)	0.85 (0.66-1.10)

* Multivariable model, hazard ratios are from multivariable Cox proportional hazard models adjusted for age, gender, TDI, ethnicity, education level, smoking status, pack-years of smoking, overall health, basal metabolic rate, physical activity level, BMI, hypertension, diabetes, depression, family history of CVD, family history of cancer, long-standing illness, cholesterol-lowering and blood pressure drug use, vitamin and mineral supplement, and intake of energy, total sugar, fresh fruit, vegetables, red meat, processed meat, alcohol, coffee, milk, NSJs, SSBs, and ASBs.

Table S8. Associations of tea consumption with cancer-caused mortality stratified by genetic caffeine metabolism score (wCMS$_{G4}$) in individuals who had genetic data.

Tea	Events/Model	Nonconsumers	Tea consumers				
			0-1.5 drinks/d	1.5-2.5 drinks/d	2.5-3.5 drinks/d	3.5-4.5 drinks/d	>4.5 drinks/d
**Unsweetened tea**							
0-2	Events, n (%)	87 (2.9)	50 (3.0)	57 (2.1)	67 (2.5)	64 (2.7)	59 (2.2)
	Multivariable model*	1 (Reference)	1.03 (0.72-1.47)	0.68 (0.48-0.96)	0.78 (0.56-1.09)	0.89 (0.63-1.27)	0.72 (0.50-1.03)
2-3	Events, n (%)	215 (3.1)	89 (2.4)	154 (2.6)	163 (2.6)	136 (2.5)	178 (2.7)
	Multivariable model*	1 (Reference)	0.84 (0.65-1.08)	0.87 (0.70-1.08)	0.87 (0.70-1.09)	0.88 (0.70-1.11)	0.95 (0.75-1.19)
3-4	Events, n (%)	282 (3.1)	114 (2.6)	220 (2.9)	196 (2.5)	170 (2.5)	243 (2.8)
	Multivariable model*	1 (Reference)	0.88 (0.71-1.10)	0.94 (0.78-1.13)	0.80 (0.66-0.98)	0.80 (0.65-0.98)	0.91 (0.74-1.10)
>4	Events, n (%)	336 (3.2)	137 (2.6)	226 (2.6)	219 (2.4)	214 (2.6)	283 (2.5)
	Multivariable model*	1 (Reference)	0.89 (0.73-1.09)	0.87 (0.73-1.03)	0.77 (0.65-0.93)	0.87 (0.72-1.04)	0.85 (0.71-1.02)
**Sugar-sweetened tea**							
0-2	Events, n (%)	87 (2.9)	11 (2.9)	18 (3.4)	16 (3.6)	14 (4.0)	11 (3.6)
	Multivariable model*	1 (Reference)	0.89 (0.46-1.72)	1.02 (0.59-1.77)	1.07 (0.59-1.91)	1.09 (0.58-2.05)	0.83 (0.40-1.71)
2-3	Events, n (%)	215 (3.1)	19 (2.4)	30 (2.8)	30 (2.9)	25 (3.3)	35 (4.5)
	Multivariable model*	1 (Reference)	0.84 (0.52-1.35)	0.95 (0.64-1.42)	0.92 (0.61-1.38)	0.93 (0.59-1.47)	1.05 (0.69-1.60)
3-4	Events, n (%)	282 (3.1)	22 (2.6)	46 (3.5)	37 (3.2)	27 (2.8)	34 (3.2)
	Multivariable model*	1 (Reference)	0.79 (0.51-1.23)	1.07 (0.77-1.49)	0.93 (0.64-1.35)	0.79 (0.52-1.22)	0.72 (0.47-1.08)
>4	Events, n (%)	336 (3.2)	25 (2.8)	50 (3.7)	55 (4.0)	40 (3.7)	56 (4.3)
	Multivariable model*	1 (Reference)	0.85 (0.56-1.28)	1.09 (0.80-1.50)	1.14 (0.84-1.55)	1.02 (0.71-1.46)	1.12 (0.80-1.57)
**Artificially sweetened tea**							
0-2	Events, n (%)	87 (2.9)	9 (6.0)	4 (1.7)	9 (4.3)	5 (3.3)	5 (3.3)
	Multivariable model*	1 (Reference)	1.87 (0.91-3.84)	0.50 (0.18-1.40)	1.13 (0.55-2.35)	0.78 (0.31-2.00)	0.86 (0.33-2.21)
2-3	Events, n (%)	215 (3.1)	10 (3.0)	20 (4.2)	16 (3.4)	18 (4.5)	21 (5.1)
	Multivariable model*	1 (Reference)	0.85 (0.45-1.61)	1.06 (0.66-1.70)	0.81 (0.48-1.37)	1.22 (0.74-2.01)	1.17 (0.72-1.90)
3-4	Events, n (%)	282 (3.1)	13 (3.4)	21 (3.5)	18 (3.0)	16 (3.3)	27 (4.3)
	Multivariable model*	1 (Reference)	0.84 (0.48-1.47)	0.90 (0.57-1.41)	0.69 (0.42-1.13)	0.85 (0.51-1.43)	0.94 (0.61-1.45)
>4	Events, n (%)	336 (3.2)	22 (4.7)	21 (3.1)	28 (4.2)	22 (3.4)	36 (4.7)
	Multivariable model*	1 (Reference)	1.18 (0.76-1.82)	0.75 (0.48-1.18)	1.08 (0.73-1.60)	0.86 (0.55-1.35)	1.16 (0.80-1.70)

* Multivariable model, hazard ratios are from multivariable Cox proportional hazard models adjusted for age, gender, TDI, ethnicity, education level, smoking status, pack-years of smoking, Overall health, Basal metabolic rate, physical activity level, BMI, hypertension, diabetes, depression, family history of CVD, family history of cancer, long-standing illness, cholesterol-lowering and blood pressure drug use, vitamin and mineral supplement, and intake of energy, total sugar, fresh fruit, vegetables, red meat, processed meat, alcohol, coffee, milk, NSJs, SSBs, and ASBs.

Table S9. Associations of tea consumption with CVD-caused mortality stratified by genetic caffeine metabolism score ($wCMS_{G4}$) in individuals who had genetic data.

Tea	Events/Model	Nonconsumers	Tea consumers				
			0-1.5 drinks/d	1.5-2.5 drinks/d	2.5-3.5 drinks/d	3.5-4.5 drinks/d	>4.5 drinks/d
**Unsweetened tea**							
0-2	Events, n (%)	28 (0.9)	11 (0.6)	17 (0.6)	26 (0.9)	18 (0.7)	14 (0.5)
	Multivariable model*	1 (Reference)	0.80 (0.39-1.63)	0.67 (0.36-1.25)	0.96 (0.54-1.71)	0.71 (0.37-1.36)	0.50 (0.24-1.01)
2-3	Events, n (%)	82 (1.1)	30 (0.8)	53 (0.9)	60 (0.9)	32 (0.6)	64 (0.9)
	Multivariable model*	1 (Reference)	0.79 (0.52-1.21)	0.84 (0.59-1.21)	0.90 (0.63-1.28)	0.58 (0.38-0.90)	0.97 (0.67-1.41)
3-4	Events, n (%)	96 (1.0)	42 (0.9)	62 (0.8)	72 (0.9)	46 (0.6)	66 (0.7)
	Multivariable model*	1 (Reference)	0.94 (0.65-1.35)	0.76 (0.55-1.06)	0.85 (0.61-1.18)	0.61 (0.42-0.89)	0.69 (0.49-0.99)
>4	Events, n (%)	138 (1.3)	61 (1.1)	74 (0.8)	82 (0.8)	80 (0.9)	83 (0.7)
	Multivariable model*	1 (Reference)	1.00 (0.73-1.36)	0.74 (0.55-0.99)	0.77 (0.58-1.04)	0.88 (0.65-1.19)	0.71 (0.53-0.97)
**Sugar-sweetened tea**							
0-2	Events, n (%)	28 (0.9)	2 (0.5)	4 (0.7)	6 (1.4)	3 (0.8)	5 (1.5)
	Multivariable model*	1 (Reference)	0.52 (0.12-2.34)	0.85 (0.27-2.70)	1.49 (0.54-4.09)	0.90 (0.24-3.34)	1.13 (0.36-3.53)
2-3	Events, n (%)	82 (1.1)	7 (0.9)	14 (1.3)	12 (1.1)	11 (1.4)	13 (1.6)
	Multivariable model*	1 (Reference)	0.72 (0.33-1.60)	1.32 (0.72-2.42)	1.08 (0.56-2.07)	1.16 (0.58-2.34)	1.04 (0.52-2.06)
3-4	Events, n (%)	96 (1.0)	14 (1.6)	16 (1.2)	15 (1.3)	10 (1.0)	18 (1.7)
	Multivariable model*	1 (Reference)	1.31 (0.73-2.37)	1.06 (0.60-1.86)	1.09 (0.60-1.98)	0.76 (0.37-1.53)	1.07 (0.58-1.97)
>4	Events, n (%)	138 (1.3)	10 (1.1)	14 (1.0)	14 (1.0)	11 (1.0)	18 (1.3)
	Multivariable model*	1 (Reference)	0.78 (0.41-1.51)	0.75 (0.42-1.33)	0.68 (0.38-1.20)	0.69 (0.35-1.33)	0.75 (0.42-1.33)
**Artificially sweetened tea**							
0-2	Events, n (%)	28 (0.9)	1 (0.7)	4 (1.7)	5 (2.2)	2 (1.3)	2 (1.3)
	Multivariable model*	1 (Reference)	0.36 (0.05-2.79)	1.11 (0.37-3.39)	1.29 (0.45-3.71)	0.64 (0.14-2.93)	0.80 (0.17-3.68)
2-3	Events, n (%)	82 (1.1)	5 (1.5)	12 (2.5)	9 (1.9)	2 (0.5)	8 (1.9)
	Multivariable model*	1 (Reference)	0.96 (0.39-2.40)	1.51 (0.80-2.83)	0.96 (0.46-1.99)	0.30 (0.07-1.26)	1.02 (0.47-2.22)
3-4	Events, n (%)	96 (1.0)	3 (0.8)	3 (0.5)	13 (2.1)	6 (1.2)	13 (2.0)
	Multivariable model*	1 (Reference)	0.58 (0.18-1.84)	0.36 (0.11-1.13)	1.33 (0.72-2.45)	0.77 (0.33-1.80)	1.09 (0.57-2.08)
>4	Events, n (%)	138 (1.3)	6 (1.3)	5 (0.7)	9 (1.3)	14 (2.2)	12 (1.5)
	Multivariable model*	1 (Reference)	0.72 (0.31-1.63)	0.43 (0.17-1.05)	0.78 (0.39-1.56)	1.32 (0.74-2.35)	0.97 (0.51-1.85)

* Multivariable model, hazard ratios are from multivariable Cox proportional hazard models adjusted for age, gender, TDI, ethnicity, education level, smoking status, pack-years of smoking, Overall health, Basal metabolic rate, physical activity level, BMI, hypertension, diabetes, depression, family history of CVD, family history of cancer, long-standing illness, cholesterol-lowering and blood pressure drug use, vitamin and mineral supplement, and intake of energy, total sugar, fresh fruit, vegetables, red meat, processed meat, alcohol, coffee, milk, NSJs, SSBs, and ASBs.

Table S10. Associations of tea consumption with all-cause mortality stratified by genetic caffeine metabolism score ($wCMS_{G4}$) in white individuals who had genetic data.

Tea	Events/Model	Nonconsumers	Tea consumers				
			0-1.5 drinks/d	1.5-2.5 drinks/d	2.5-3.5 drinks/d	3.5-4.5 drinks/d	>4.5 drinks/d
**Unsweetened tea**							
0-2	Events, n (%)	197 (6.6)	88 (5.3)	139 (5.0)	172 (6.1)	131 (5.4)	135 (4.7)
	Multivariable model*	1 (Reference)	0.87 (0.67-1.12)	0.79 (0.63-0.99)	0.93 (0.74-1.15)	0.83 (0.65-1.05)	0.77 (0.60-0.98)
2-3	Events, n (%)	488 (6.9)	215 (5.7)	344 (5.6)	362 (5.4)	300 (5.3)	395 (5.6)
	Multivariable model*	1 (Reference)	0.93 (0.79-1.10)	0.89 (0.77-1.02)	0.88 (0.76-1.01)	0.86 (0.74-1.00)	0.91 (0.78-1.06)
3-4	Events, n (%)	637 (6.8)	245 (5.3)	432 (5.4)	459 (5.4)	361 (4.9)	522 (5.5)
	Multivariable model*	1 (Reference)	0.88 (0.75-1.02)	0.85 (0.75-0.96)	0.86 (0.76-0.98)	0.76 (0.66-0.87)	0.87 (0.76-0.99)
>4	Events, n (%)	822 (7.4)	350 (6.4)	510 (5.5)	525 (5.3)	479 (5.3)	650 (5.3)
	Multivariable model*	1 (Reference)	0.96 (0.84-1.09)	0.82 (0.73-0.92)	0.77 (0.69-0.87)	0.79 (0.70-0.89)	0.80 (0.71-0.90)
**Sugar-sweetened tea**							
0-2	Events, n (%)	197 (6.6)	19 (6.1)	31 (7.5)	31 (8.0)	19 (5.6)	24 (7.9)
	Multivariable model*	1 (Reference)	0.79 (0.49-1.29)	0.96 (0.64-1.44)	1.02 (0.67-1.54)	0.73 (0.44-1.22)	0.85 (0.52-1.39)
2-3	Events, n (%)	488 (6.9)	39 (6.0)	64 (6.9)	70 (7.3)	51 (6.8)	77 (9.5)
	Multivariable model*	1 (Reference)	0.76 (0.54-1.06)	0.90 (0.69-1.18)	0.97 (0.74-1.27)	0.83 (0.61-1.14)	0.93 (0.70-1.24)
3-4	Events, n (%)	637 (6.8)	54 (6.9)	108 (8.6)	74 (6.5)	77 (7.8)	94 (8.5)
	Multivariable model*	1 (Reference)	0.84 (0.63-1.11)	1.15 (0.93-1.43)	0.81 (0.63-1.05)	0.90 (0.69-1.16)	0.79 (0.61-1.02)
>4	Events, n (%)	822 (7.4)	57 (6.4)	96 (7.0)	115 (8.2)	84 (7.4)	134 (9.3)
	Multivariable model*	1 (Reference)	0.81 (0.62-1.07)	0.86 (0.69-1.07)	0.94 (0.76-1.16)	0.80 (0.63-1.03)	0.90 (0.72-1.12)
**Artificially sweetened tea**							
0-2	Events, n (%)	197 (6.6)	16 (11.6)	13 (6.1)	25 (11.9)	12 (7.6)	18 (10.6)
	Multivariable model*	1 (Reference)	1.09 (0.64-1.85)	0.71 (0.40-1.26)	1.25 (0.80-1.95)	0.75 (0.41-1.37)	1.01 (0.60-1.70)
2-3	Events, n (%)	488 (6.9)	24 (7.1)	55 (11.3)	46 (9.4)	37 (8.7)	44 (9.9)
	Multivariable model*	1 (Reference)	0.85 (0.56-1.28)	1.19 (0.89-1.58)	0.93 (0.68-1.27)	1.07 (0.76-1.51)	0.96 (0.69-1.34)
3-4	Events, n (%)	637 (6.8)	29 (7.6)	58 (9.2)	52 (8.1)	39 (7.4)	71 (10.2)
	Multivariable model*	1 (Reference)	0.81 (0.55-1.17)	1.03 (0.79-1.36)	0.83 (0.62-1.11)	0.82 (0.59-1.14)	0.92 (0.70-1.21)
>4	Events, n (%)	822 (7.4)	48 (9.8)	45 (6.3)	71 (9.7)	64 (9.3)	74 (8.9)
	Multivariable model*	1 (Reference)	1.03 (0.77-1.38)	0.62 (0.46-0.84)	0.95 (0.74-1.22)	0.86 (0.66-1.12)	0.86 (0.66-1.11)

* Multivariable model, estimates are hazard ratios from multivariable Cox proportional hazard models adjusted for age, gender, TDI, education level, smoking status, pack-years of smoking, Overall health, Basal metabolic rate, physical activity level, BMI, hypertension, diabetes, depression, family history of CVD, family history of cancer, long-standing illness, cholesterol-lowering and blood pressure drug use, vitamin and mineral supplement, and intake of energy, total sugar, fresh fruit, vegetables, red meat, processed meat, alcohol, coffee, milk, NSJs, SSBs, and ASBs.

Table S11. Associations of tea consumption with cancer mortality stratified by genetic caffeine metabolism score ($wCMS_{G4}$) in white individuals who had genetic data.

Tea	Events/Model	Nonconsumers	Tea consumers				
			0-1.5 drinks/d	1.5-2.5 drinks/d	2.5-3.5 drinks/d	3.5-4.5 drinks/d	>4.5 drinks/d
**Unsweetened tea**							
0-2	Events, n (%)	81 (3.0)	48 (3.1)	56 (2.2)	64 (2.5)	63 (2.8)	57 (2.2)
	Multivariable model*	1 (Reference)	1.05 (0.73-1.51)	0.71 (0.50-1.01)	0.78 (0.55-1.11)	0.91 (0.64-1.31)	0.73 (0.50-1.06)
2-3	Events, n (%)	208 (3.2)	84 (2.4)	149 (2.7)	159 (2.6)	133 (2.6)	174 (2.7)
	Multivariable model*	1 (Reference)	0.82 (0.63-1.05)	0.87 (0.70-1.08)	0.87 (0.70-1.09)	0.87 (0.69-1.11)	0.94 (0.75-1.18)
3-4	Events, n (%)	269 (3.1)	111 (2.6)	214 (2.9)	192 (2.5)	168 (2.5)	239 (2.8)
	Multivariable model*	1 (Reference)	0.90 (0.72-1.13)	0.96 (0.79-1.15)	0.82 (0.67-1.00)	0.82 (0.67-1.01)	0.93 (0.76-1.13)
>4	Events, n (%)	329 (3.2)	136 (2.7)	222 (2.6)	218 (2.4)	210 (2.6)	281 (2.5)
	Multivariable model*	1 (Reference)	0.90 (0.73-1.10)	0.87 (0.73-1.03)	0.78 (0.65-0.94)	0.86 (0.71-1.04)	0.86 (0.72-1.03)
**Sugar-sweetened tea**							
0-2	Events, n (%)	81 (3.0)	9 (3.2)	16 (4.2)	15 (4.1)	11 (3.6)	10 (3.6)
	Multivariable model*	1 (Reference)	0.90 (0.44-1.84)	1.12 (0.63-2.01)	1.20 (0.65-2.21)	1.08 (0.54-2.19)	0.88 (0.41-1.89)
2-3	Events, n (%)	208 (3.2)	18 (3.0)	28 (3.2)	27 (3.0)	22 (3.2)	35 (4.7)
	Multivariable model*	1 (Reference)	0.87 (0.53-1.42)	0.95 (0.63-1.44)	0.86 (0.56-1.33)	0.85 (0.53-1.37)	1.06 (0.69-1.62)
3-4	Events, n (%)	269 (3.1)	21 (2.9)	46 (4.0)	34 (3.2)	26 (2.9)	32 (3.2)
	Multivariable model*	1 (Reference)	0.81 (0.52-1.28)	1.17 (0.84-1.63)	0.91 (0.62-1.34)	0.80 (0.51-1.23)	0.70 (0.46-1.08)
>4	Events, n (%)	329 (3.2)	24 (3.0)	46 (3.7)	51 (3.9)	40 (3.9)	56 (4.4)
	Multivariable model*	1 (Reference)	0.87 (0.57-1.33)	1.06 (0.76-1.47)	1.09 (0.79-1.50)	1.04 (0.73-1.50)	1.13 (0.81-1.59)
**Artificially sweetened tea**							
0-2	Events, n (%)	81 (3.0)	8 (6.5)	4 (2.0)	9 (4.8)	5 (3.6)	4 (2.8)
	Multivariable model*	1 (Reference)	2.03 (0.95-4.36)	0.59 (0.21-1.64)	1.31 (0.63-2.74)	0.84 (0.33-2.18)	0.73 (0.25-2.08)
2-3	Events, n (%)	208 (3.2)	10 (3.2)	18 (4.1)	13 (3.0)	17 (4.5)	19 (4.7)
	Multivariable model*	1 (Reference)	0.89 (0.47-1.69)	1.01 (0.62-1.65)	0.67 (0.38-1.20)	1.17 (0.70-1.96)	1.05 (0.63-1.73)
3-4	Events, n (%)	269 (3.1)	13 (3.7)	20 (3.5)	17 (3.0)	16 (3.4)	27 (3.4)
	Multivariable model*	1 (Reference)	0.89 (0.51-1.56)	0.91 (0.57-1.45)	0.68 (0.41-1.13)	0.90 (0.54-1.52)	0.97 (0.63-1.50)
>4	Events, n (%)	329 (3.2)	22 (4.9)	21 (3.2)	27 (4.1)	22 (3.5)	36 (4.7)
	Multivariable model*	1 (Reference)	1.21 (0.78-1.87)	0.77 (0.49-1.21)	1.06 (0.71-1.59)	0.88 (0.56-1.38)	1.18 (0.81-1.73)

* Multivariable model, estimates are hazard ratios from multivariable Cox proportional hazard models adjusted for age, gender, TDI, education level, smoking status, pack-years of smoking, Overall health, Basal metabolic rate, physical activity level, BMI, hypertension, diabetes, depression, family history of CVD, family history of cancer, long-standing illness, cholesterol-lowering and blood pressure drug use, vitamin and mineral supplement, and intake of energy, total sugar, fresh fruit, vegetables, red meat, processed meat, alcohol, coffee, milk, NSJs, SSBs, and ASBs.

Table S12. Associations of tea consumption with CVD mortality stratified by genetic caffeine metabolism score ($wCMS_{G4}$) in white individuals who had genetic data.

Tea	Events/Model	Nonconsumers	Tea consumers				
			0-1.5 drinks/d	1.5-2.5 drinks/d	2.5-3.5 drinks/d	3.5-4.5 drinks/d	>4.5 drinks/d
**Unsweetened tea**							
0-2	Events, n (%)	25 (0.9)	10 (0.6)	16 (0.6)	25 (0.9)	18 (0.8)	14 (0.5)
	Multivariable model*	1 (Reference)	0.78 (0.37-1.66)	0.68 (0.36-1.31)	0.99 (0.54-1.80)	0.75 (0.39-1.46)	0.52 (0.25-1.07)
2-3	Events, n (%)	76 (1.1)	29 (0.8)	53 (0.9)	56 (0.9)	30 (0.5)	63 (0.9)
	Multivariable model*	1 (Reference)	0.81 (0.53-1.26)	0.89 (0.61-1.28)	0.88 (0.61-1.27)	0.57 (0.37-0.90)	1.00 (0.68-1.46)
3-4	Events, n (%)	92 (1.0)	39 (0.9)	62 (0.8)	69 (0.8)	45 (0.6)	65 (0.7)
	Multivariable model*	1 (Reference)	0.89 (0.61-1.30)	0.77 (0.55-1.08)	0.82 (0.59-1.15)	0.60 (0.41-0.88)	0.68 (0.48-0.97)
>4	Events, n (%)	134 (1.3)	58 (1.1)	72 (0.8)	82 (0.9)	79 (0.9)	82 (0.7)
	Multivariable model*	1 (Reference)	0.97 (0.71-1.33)	0.73 (0.54-0.99)	0.78 (0.59-1.05)	0.87 (0.64-1.18)	0.71 (0.52-0.97)
**Sugar-sweetened tea**							
0-2	Events, n (%)	25 (0.9)	2 (0.7)	4 (1.0)	6 (1.6)	2 (0.6)	2 (0.7)
	Multivariable model*	1 (Reference)	0.63 (0.14-2.86)	0.99 (0.30-3.25)	1.62 (0.58-4.55)	0.69 (0.15-3.27)	0.44 (0.09-2.19)
2-3	Events, n (%)	76 (1.1)	6 (1.0)	11 (1.3)	12 (1.3)	9 (1.3)	13 (1.7)
	Multivariable model*	1 (Reference)	0.72 (0.31-1.68)	1.16 (0.60-2.26)	1.16 (0.60-2.25)	0.98 (0.46-2.10)	1.01 (0.50-2.03)
3-4	Events, n (%)	92 (1.0)	12 (1.6)	15 (1.3)	14 (1.3)	10 (1.1)	18 (1.7)
	Multivariable model*	1 (Reference)	1.24 (0.66-2.33)	1.11 (0.62-1.98)	1.10 (0.60-2.04)	0.81 (0.40-1.64)	1.13 (0.61-2.11)
>4	Events, n (%)	134 (1.3)	10 (1.2)	13 (1.0)	13 (1.0)	22 (1.0)	18 (1.3)
	Multivariable model*	1 (Reference)	0.84 (0.44-1.62)	0.72 (0.40-1.30)	0.63 (0.35-1.15)	0.69 (0.36-1.35)	0.74 (0.41-1.32)
**Artificially sweetened tea**							
0-2	Events, n (%)	25 (0.9)	0 (0)	4 (2.0)	5 (2.5)	1 (0.7)	2 (1.3)
	Multivariable model*	1 (Reference)	0	1.40 (0.46-4.30)	1.44 (0.48-4.27)	0.33 (0.04-2.66)	0.81 (0.17-3.82)
2-3	Events, n (%)	76 (1.1)	5 (1.6)	12 (2.7)	9 (2.0)	1 (0.2)	8 (2.0)
	Multivariable model*	1 (Reference)	1.06 (0.42-2.65)	1.64 (0.87-3.10)	1.07 (0.51-2.22)	0.16 (0.02-1.15)	1.04 (0.47-2.27)
3-4	Events, n (%)	92 (1.0)	3 (0.8)	3 (0.5)	13 (2.2)	4 (0.8)	13 (2.1)
	Multivariable model*	1 (Reference)	0.62 (0.19-1.98)	0.38 (0.12-1.20)	1.39 (0.75-2.57)	0.53 (0.19-1.48)	1.06 (0.55-2.03)
>4	Events, n (%)	134 (1.3)	4 (0.9)	5 (0.7)	8 (1.2)	14 (2.2)	12 (1.5)
	Multivariable model*	1 (Reference)	0.51 (0.19-1.38)	0.45 (0.18-1.10)	0.73 (0.35-1.51)	1.35 (0.75-2.42)	0.99 (0.52-1.88)

* Multivariable model, estimates are hazard ratios from multivariable Cox proportional hazard models adjusted for age, gender, TDI, education level, smoking status, pack-years of smoking, Overall health, Basal metabolic rate, physical activity level, BMI, hypertension, diabetes, depression, family history of CVD, family history of cancer, long-standing illness, cholesterol-lowering and blood pressure drug use, vitamin and mineral supplement, and intake of energy, total sugar, fresh fruit, vegetables, red meat, processed meat, alcohol, coffee, milk, NSJs, SSBs, and ASBs.

Table S13. Associations of standard tea (black tea) consumption with risk of all-cause and cause-specific mortality.

Outcome	Events/Model	Nonconsumers	Tea consumers				
			0-1.5 drinks/d	1.5-2.5 drinks/d	2.5-3.5 drinks/d	3.5-4.5 drinks/d	>4.5 drinks/d
**Unsweetened tea**							
All-cause mortality	Events, n (%)	3135 (6.3)	1056 (5.3)	1602 (5.3)	1454 (5.4)	1130 (5.5)	1129 (5.7)
(n = 166918)	Multivariable model*	1 (Reference)	0.91 (0.85-0.98)	0.89 (0.84-0.95)	0.87 (0.82-0.93)	0.86 (0.8-0.93)	0.85 (0.79-0.92)
Cancer mortality	Events, n (%)	1293 (2.8)	463 (2.5)	704 (2.6)	596 (2.4)	534 (2.9)	500 (2.8)
(n = 152086)	Multivariable model*	1 (Reference)	0.93 (0.83-1.03)	0.91 (0.83-1)	0.85 (0.77-0.94)	0.98 (0.88-1.09)	0.93 (0.83-1.04)
CVD mortality	Events, n (%)	467 (1.0)	154 (0.8)	229 (0.8)	221 (0.9)	152 (0.8)	154 (0.8)
(n = 160231)	Multivariable model*	1 (Reference)	0.88 (0.73-1.06)	0.85 (0.72-0.99)	0.89 (0.76-1.06)	0.79 (0.65-0.96)	0.80 (0.66-0.98)
**Sugar-sweetened tea**							
All-cause mortality	Events, n (%)	3135 (6.3)	199 (5.7)	330 (6.8)	294 (7.0)	245 (7.6)	299 (9.2)
(n =69011)	Multivariable model*	1 (Reference)	0.83 (0.72-0.96)	0.96 (0.85-1.08)	0.93 (0.82-1.06)	0.93 (0.81-1.07)	0.93 (0.81-1.06)
Cancer mortality	Events, n (%)	1293 (2.8)	83 (2.6)	141 (3.2)	130 (3.3)	108 (3.7)	123 (4.2)
(n =63100)	Multivariable model*	1 (Reference)	0.86 (0.69-1.08)	1 (0.84-1.2)	1.03 (0.85-1.24)	1.07 (0.87-1.32)	1.05 (0.85-1.29)
CVD mortality	Events, n (%)	467 (1.0)	34 (1.0)	56 (1.2)	45 (1.1)	32 (1.1)	44 (1.4)
(n = 65885)	Multivariable model*	1 (Reference)	0.91 (0.64-1.3)	1.08 (0.81-1.44)	0.95 (0.69-1.31)	0.83 (0.57-1.22)	0.9 (0.64-1.28)
**Artificially sweetened tea**							
All-cause mortality	Events, n (%)	3135 (6.3)	131 (8.3)	196 (8.6)	191 (9.0)	146 (8.7)	181 (10.0)
(n = 63100)	Multivariable model*	1 (Reference)	0.95 (0.80-1.13)	0.97 (0.83-1.12)	0.97 (0.83-1.12)	0.93 (0.78-1.1)	0.97 (0.82-1.13)
Cancer mortality	Events, n (%)	1293 (2.8)	60 (4.2)	72 (3.5)	63 (3.3)	58 (3.8)	74 (4.5)
(n = 54223)	Multivariable model*	1 (Reference)	1.13 (0.87-1.46)	0.93 (0.73-1.18)	0.87 (0.68-1.13)	1.04 (0.79-1.36)	1.11 (0.87-1.42)
CVD mortality	Events, n (%)	467 (1.0)	16 (1.1)	26 (1.2)	34 (1.8)	23 (1.5)	31 (1.9)
(n = 56582)	Multivariable model*	1 (Reference)	0.75 (0.46-1.24)	0.83 (0.56-1.23)	1.1 (0.77-1.57)	0.9 (0.58-1.38)	1.05 (0.72-1.54)

Note: CVD, cardiovascular disease. one drink is equal to approximately 250 mL or 8.5 ounces.

* Multivariable model, estimates are hazard ratios (95% CIs) from multivariable Cox proportional hazard models adjusted for age (continuous), gender, Townsend deprivation index (continuous), education level (degree or no degree), ethnicity (white or other), smoking status (current, former, or never), pack-years of smoking (continuous), Overall health (poor, fair, good, or excellent), Basal metabolic rate (continuous), physical activity level (low, moderate, or high), body mass index (continuous), hypertension (yes or no), diabetes (yes or no), depression (yes or no), family history of CVD disease (yes or no), family history of cancer (yes or no), long-standing illness (yes or no), cholesterol-lowering drug use (yes or no), blood pressure drug use (yes or no), vitamin and mineral supplement (yes or no), and intake of energy, total sugar, fresh fruit, vegetables, red meat, processed meat, alcohol, coffee, milk, naturally sweet juices, sugar-sweetened beverages, and artificially sweetened beverages.

Table S14. Associations of tea consumption with all-cause and cause-specific mortality after excluding participants with missing covariates.

Outcome	Events/Model	Nonconsumers	Tea consumers				
			0-1.5 drinks/d	1.5-2.5 drinks/d	2.5-3.5 drinks/d	3.5-4.5 drinks/d	>4.5 drinks/d
**Unsweetened tea**							
All-cause mortality	Events, n (%)	1395 (6.5)	629 (5.7)	986 (5.4)	989 (5.1)	809 (4.8)	1038 (4.8)
(n = 109147)	Multivariable model*	1 (Reference)	0.96 (0.87-1.05)	0.88 (0.80-0.95)	0.83 (0.76-0.90)	0.77 (0.70-0.85)	0.79 (0.72-0.86)
Cancer mortality	Events, n (%)	601 (3.0)	262 (2.6)	460 (2.7)	440 (2.5)	390 (2.5)	465 (2.4)
(n = 99819)	Multivariable model*	1 (Reference)	0.89 (0.77-1.03)	0.92 (0.81-1.04)	0.83 (0.73-0.95)	0.85 (0.74-0.98)	0.81 (0.71-0.93)
CVD mortality	Events, n (%)	217 (1.1)	99 (0.9)	134 (0.8)	146 (0.8)	96 (0.6)	136 (0.7)
(n = 105167)	Multivariable model*	1 (Reference)	0.97 (0.76-1.24)	0.78 (0.62-0.98)	0.81 (0.65-1.02)	0.63 (0.49-0.81)	0.74 (0.58-0.95)
**Sugar-sweetened tea**							
All-cause mortality	Events, n (%)	1395 (6.5)	124 (5.9)	198 (6.5)	200 (7.1)	141 (6.3)	193 (8.1)
(n = 34176)	Multivariable model*	1 (Reference)	0.84 (0.69-1.01)	0.92 (0.79-1.08)	0.94 (0.80-1.11)	0.79 (0.66-0.96)	0.84 (0.70-1.00)
Cancer mortality	Events, n (%)	601 (3.0)	54 (2.7)	94 (3.4)	86 (3.3)	64 (3.2)	74 (3.4)
(n = 31523)	Multivariable model*	1 (Reference)	0.88 (0.66-1.17)	1.03 (0.82-1.30)	0.98 (0.77-1.25)	0.92 (0.69-1.21)	0.84 (0.64-1.11)
CVD mortality	Events, n (%)	217 (1.1)	21 (1.0)	34 (1.2)	27 (1.0)	21 (1.0)	30 (1.3)
(n = 32663)	Multivariable model*	1 (Reference)	0.85 (0.54-1.35)	0.99 (0.67-1.44)	0.82 (0.54-1.25)	0.78 (0.48-1.27)	0.87 (0.56-1.36)
**Artificially sweetened tea**							
All-cause mortality	Events, n (%)	1395 (6.5)	82 (8.3)	111 (7.8)	113 (8.1)	94 (8.0)	120 (8.7)
(n = 27971)	Multivariable model*	1 (Reference)	1.00 (0.80-1.25)	0.84 (0.69-1.02)	0.87 (0.71-1.06)	0.86 (0.69-1.07)	0.87 (0.71-1.07)
Cancer mortality	Events, n (%)	601 (3.0)	39 (4.3)	35 (2.7)	41 (3.2)	40 (3.8)	45 (3.6)
(n = 25722)	Multivariable model*	1 (Reference)	1.16 (0.83-1.60)	0.67 (0.47-0.95)	0.83 (0.60-1.14)	0.98 (0.71-1.37)	0.89 (0.64-1.23)
CVD mortality	Events, n (%)	217 (1.1)	9 (1.0)	14 (1.1)	20 (1.6)	12 (1.1)	24 (1.9)
(n = 26563)	Multivariable model*	1 (Reference)	0.70 (0.36-1.38)	0.71 (0.41-1.23)	1.02 (0.64-1.65)	0.72 (0.40-1.31)	1.18 (0.74-1.87)

Note: CVD, cardiovascular disease. one drink is equal to approximately 250 mL or 8.5 ounces.

* Multivariable model, estimates are hazard ratios (95% CIs) from multivariable Cox proportional hazard models adjusted for age (continuous), gender, Townsend deprivation index (continuous), education level (degree or no degree), ethnicity (white or other), smoking status (current, former, or never), pack-years of smoking (continuous), Overall health (poor, fair, good, or excellent), Basal metabolic rate (continuous), physical activity level (low, moderate, or high), body mass index (continuous), hypertension (yes or no), diabetes (yes or no), depression (yes or no), family history of CVD disease (yes or no), family history of cancer (yes or no), long-standing illness (yes or no), cholesterol-lowering drug use (yes or no), blood pressure drug use (yes or no), vitamin and mineral supplement (yes or no), and intake of energy, total sugar, fresh fruit, vegetables, red meat, processed meat, alcohol, coffee, milk, naturally sweet juices, sugar-sweetened beverages, and artificially sweetened beverages.

Table S15. Associations of tea consumption with all-cause and cause-specific mortality after excluding participants who had an outcome event during the first two years of follow-up.

Outcome	Events/Model	Nonconsumers	Tea consumers				
			0-1.5 drinks/d	1.5-2.5 drinks/d	2.5-3.5 drinks/d	3.5-4.5 drinks/d	>4.5 drinks/d
**Unsweetened tea**							
All-cause mortality	Events, n (%)	2215 (6.8)	933 (5.6)	1465 (5.3)	1550 (5.3)	1303 (5.1)	1702 (5.2)
(n = 165268)	Multivariable model*	1 (Reference)	0.93 (0.86-1.01)	0.84 (0.79-0.90)	0.84 (0.79-0.90)	0.81 (0.75-0.87)	0.83 (0.78-0.90)
Cancer mortality	Events, n (%)	916 (3.0)	396 (2.6)	661 (2.6)	645 (2.4)	596 (2.6)	759 (2.5)
(n = 150732)	Multivariable model*	1 (Reference)	0.91 (0.81-1.03)	0.89 (0.80-0.98)	0.82 (0.73-0.91)	0.88 (0.78-0.98)	0.88 (0.79-0.98)
CVD mortality	Events, n (%)	339 (1.1)	144 (0.9)	209 (0.8)	240 (0.8)	178 (0.7)	224 (0.7)
(n = 158850)	Multivariable model*	1 (Reference)	0.93 (0.76-1.14)	0.79 (0.66-0.94)	0.86 (0.72-1.03)	0.75 (0.62-0.91)	0.76 (0.63-0.92)
**Sugar-sweetened tea**							
All-cause mortality	Events, n (%)	2215 (6.8)	186 (5.8)	317 (6.7)	304 (7.0)	240 (6.8)	329 (8.4)
(n = 52484)	Multivariable model*	1 (Reference)	0.81 (0.70-0.95)	0.96 (0.85-1.09)	0.93 (0.82-1.06)	0.85 (0.74-0.99)	0.88 (0.77-1.01)
Cancer mortality	Events, n (%)	916 (3.0)	78 (2.6)	140 (3.2)	137 (3.4)	107 (3.4)	131 (3.7)
(n = 48264)	Multivariable model*	1 (Reference)	0.84 (0.67-1.07)	1.03 (0.85-1.24)	1.02 (0.84-1.24)	0.97 (0.78-1.21)	0.93 (0.75-1.15)
CVD mortality	Events, n (%)	339 (1.1)	35 (1.1)	47 (1.1)	46 (1.1)	32 (1.0)	53 (1.4)
(n = 49959)	Multivariable model*	1 (Reference)	0.96 (0.67-1.37)	0.94 (0.68-1.30)	0.93 (0.67-1.30)	0.80 (0.54-1.18)	0.98 (0.70-1.39)
**Artificially sweetened tea**							
All-cause mortality	Events, n (%)	2215 (6.8)	123 (8.3)	182 (8.1)	201 (9.1)	161 (8.4)	215 (9.6)
(n = 42865)	Multivariable model*	1 (Reference)	0.93 (0.77-1.12)	0.90 (0.77-1.04)	0.96 (0.83-1.11)	0.92 (0.78-1.09)	0.97 (0.83-1.13)
Cancer mortality	Events, n (%)	916 (3.0)	53 (3.9)	67 (3.3)	71 (3.6)	62 (3.6)	91 (4.5)
(n = 39277)	Multivariable model*	1 (Reference)	1.02 (0.77-1.35)	0.86 (0.67-1.10)	0.92 (0.72-1.18)	0.98 (0.75-1.27)	1.13 (0.89-1.42)
CVD mortality	Events, n (%)	339 (1.1)	15 (1.1)	24 (1.1)	35 (1.7)	23 (1.3)	37 (1.8)
(n = 40548)	Multivariable model*	1 (Reference)	0.73 (0.43-1.23)	0.77 (0.51-1.17)	1.08 (0.75-1.55)	0.84 (0.55-1.31)	1.10 (0.76-1.60)

Note: CVD, cardiovascular disease. one drink is equal to approximately 250 mL or 8.5 ounces.

* Multivariable model, estimates are hazard ratios (95% CIs) from multivariable Cox proportional hazard models adjusted for age (continuous), gender, Townsend deprivation index (continuous), education level (degree or no degree), ethnicity (white or other), smoking status (current, former, or never), pack-years of smoking (continuous), Overall health (poor, fair, good, or excellent), Basal metabolic rate (continuous), physical activity level (low, moderate, or high), body mass index (continuous), hypertension (yes or no), diabetes (yes or no), depression (yes or no), family history of CVD disease (yes or no), family history of cancer (yes or no), long-standing illness (yes or no), cholesterol-lowering drug use (yes or no), blood pressure drug use (yes or no), vitamin and mineral supplement (yes or no), and intake of energy, total sugar, fresh fruit, vegetables, red meat, processed meat, alcohol, coffee, milk, naturally sweet juices, sugar-sweetened beverages, and artificially sweetened beverages.

Table S16. Associations of tea consumption with all-cause and cause-specific mortality after removing sugar added to tea from total sugar.

Outcome	Events/Model	Nonconsumers	Tea consumers				
			0-1.5 drinks/d	1.5-2.5 drinks/d	2.5-3.5 drinks/d	3.5-4.5 drinks/d	>4.5 drinks/d
**Unsweetened tea**							
All-cause mortality	Events, n (%)	2286 (7.0)	946 (5.7)	1493 (5.4)	1593 (5.4)	1333 (5.2)	1572 (5.3)
(n = 165503)	Multivariable model*	1 (Reference)	0.92 (0.85-0.99)	0.83 (0.78-0.89)	0.84 (0.78-0.90)	0.80 (0.75-0.86)	0.83 (0.77-0.89)
Cancer mortality	Events, n (%)	941 (3.1)	401 (2.6)	668 (2.6)	658 (2.5	606 (2.6)	774 (2.6)
(n = 150807)	Multivariable model*	1 (Reference)	0.90 (0.80-1.01)	0.87 (0.78-0.96)	0.81 (0.73-0.90)	0.86 (0.77-0.97)	0.87 (0.78-0.97)
CVD mortality	Events, n (%)	350 (1.1)	147 (0.9)	213 (0.8)	244 (0.9)	179 (0.7)	228 (0.7)
(n = 158877)	Multivariable model*	1 (Reference)	0.93 (0.76-1.13)	0.78 (0.66-0.93)	0.85 (0.72-1.02)	0.73 (0.60-0.89)	0.75 (0.62-0.91)
**Sugar-sweetened tea**							
All-cause mortality	Events, n (%)	2286 (7.0)	192 (5.9)	326 (6.9)	310 (7.1)	251 (7.1)	339 (8.6)
(n = 52597)	Multivariable model*	1 (Reference)	0.82 (0.70-0.95)	0.96 (0.85-1.08)	0.92 (0.81-1.04)	0.86 (0.75-0.99)	0.87 (0.77-1.00)
Cancer mortality	Events, n (%)	941 (3.1)	80 (2.7)	144 (3.3)	139 (3.4)	108 (3.4)	136 (3.8)
(n = 48303)	Multivariable model*	1 (Reference)	0.85 (0.67-1.07)	1.03 (0.86-1.24)	1.01 (0.83-1.22)	0.95 (0.77-1.18)	0.93 (0.76-1.15)
CVD mortality	Events, n (%)	350 (1.1)	35 (1.1)	49 (1.1)	47 (1.1)	35 (1.1)	54 (1.5)
(n = 49977)	Multivariable model*	1 (Reference)	0.93 (0.65-1.32)	0.95 (0.69-1.30)	0.92 (0.66-1.27)	0.84 (0.58-1.22)	0.96 (0.68-1.34)
**Artificially sweetened tea**							
All-cause mortality	Events, n (%)	2286 (7.0)	125 (8.4)	187 (8.3)	204 (9.2)	164 (8.6)	217 (9.7)
(n = 42951)	Multivariable model*	1 (Reference)	0.91 (0.76-1.10)	0.89 (0.76-1.03)	0.94(0.81-1.09)	0.90 (0.77-1.07)	0.94 (0.81-1.09)
Cancer mortality	Events, n (%)	941 (3.1)	54 (3.9)	68 (3.3)	71 (3.6)	63 (3.7)	91 (4.5)
(n = 39305)	Multivariable model*	1 (Reference)	1.01 (0.77-1.33)	0.84 (0.66-1.08)	0.89 (0.69-1.14)	0.96 (0.74-1.25)	1.09 (0.86-1.37)
CVD mortality	Events, n (%)	350 (1.1)	15 (1.1)	25 (1.2)	36 (1.8)	24 (1.4)	37 (1.8)
(n = 40562)	Multivariable model*	1 (Reference)	0.71 (0.42-1.19)	0.78 (0.51-1.17)	1.07 (0.75-1.53)	0.85 (0.55-1.30)	1.05 (0.73-1.52)

Note: CVD, cardiovascular disease. one drink is equal to approximately 250 mL or 8.5 ounces.

* Multivariable model, estimates are hazard ratios (95% CIs) from multivariable Cox proportional hazard models adjusted for age (continuous), gender, Townsend deprivation index (continuous), education level (degree or no degree), ethnicity (white or other), smoking status (current, former, or never), pack-years of smoking (continuous), Overall health (poor, fair, good, or excellent), Basal metabolic rate (continuous), physical activity level (low, moderate, or high), body mass index (continuous), hypertension (yes or no), diabetes (yes or no), depression (yes or no), family history of CVD disease (yes or no), family history of cancer (yes or no), long-standing illness (yes or no), cholesterol-lowering drug use (yes or no), blood pressure drug use (yes or no), vitamin and mineral supplement (yes or no), and intake of energy, total sugar, fresh fruit, vegetables, red meat, processed meat, alcohol, coffee, milk, naturally sweet juices, sugar-sweetened beverages, and artificially sweetened beverages.

Table S17. Associations of tea consumption with all-cause and cause-specific mortality after excluding participants who were drinking tea last year but not drinking tea the day before.

Outcome	Events/Model	Nonconsumers	Tea consumers				
			0-1.5 drinks/d	1.5-2.5 drinks/d	2.5-3.5 drinks/d	3.5-4.5 drinks/d	>4.5 drinks/d
**Unsweetened tea**							
All-cause mortality	Events, n (%)	2286 (7.0)	825 (5.8)	1424 (5.4)	1552 (5.4)	1314 (5.2)	1720 (5.3)
(n = 159617)	Multivariable model*	1 (Reference)	0.91 (0.84-0.99)	0.83 (0.78-0.89)	0.84 (0.78-0.89)	0.80 (0.75-0.86)	0.82 (0.77-0.88)
Cancer mortality	Events, n (%)	941 (3.1)	353 (2.7)	626 (2.6)	635 (2.4)	597 (2.6)	761 (2.6)
(n = 145418)	Multivariable model*	1 (Reference)	0.91 (0.80-1.03)	0.86 (0.77-0.95)	0.81 (0.73-0.90)	0.88 (0.78-0.98)	0.88 (0.79-0.98)
CVD mortality	Events, n (%)	350 (1.1)	124 (0.9)	210 (0.8)	241 (0.9)	178 (0.7)	225 (0.7)
(n = 153172)	Multivariable model*	1 (Reference)	0.89 (0.72-1.10)	0.81 (0.68-0.97)	0.87 (0.73-1.04)	0.75 (0.62-0.91)	0.76 (0.63-0.92)
**Sugar-sweetened tea**							
All-cause mortality	Events, n (%)	2286 (7.0)	171 (5.9)	316 (6.9)	304 (7.1)	251 (7.2)	333 (8.6)
(n = 51986)	Multivariable model*	1 (Reference)	0.80 (0.68-0.94)	0.95 (0.84-1.08)	0.91 (0.80-1.03)	0.86 (0.75-0.99)	0.86 (0.75-0.99)
Cancer mortality	Events, n (%)	941 (3.1)	69 (2.6)	142 (3.4)	137 (3.4)	108 (3.4)	133 (3.8)
(n = 47744)	Multivariable model*	1 (Reference)	0.81 (0.63-1.04)	1.05 (0.87-1.26)	1.00 (0.83-1.22)	0.96 (0.77-1.19)	0.92 (0.75-1.14)
CVD mortality	Events, n (%)	350 (1.1)	32 (1.1)	45 (1.0)	46 (1.1)	35 (1.1)	53 (1.4)
(n = 49398)	Multivariable model*	1 (Reference)	0.93 (0.64-1.35)	0.89 (0.64-1.24)	0.91 (0.66-1.27)	0.85 (0.58-1.24)	0.95 (0.68-1.34)
**Artificially sweetened tea**							
All-cause mortality	Events, n (%)	2286 (7.0)	110 (8.2)	180 (8.3)	197 (9.2)	162 (8.6)	214 (9.7)
(n = 42588)	Multivariable model*	1 (Reference)	0.88 (0.73-1.07)	0.89 (0.76-1.03)	0.93 (0.80-1.08)	0.91 (0.77-1.07)	0.94 (0.80-1.09)
Cancer mortality	Events, n (%)	941 (3.1)	48 (3.9)	65 (3.3)	70 (3.6)	63 (3.7)	88 (4.4)
(n = 38983)	Multivariable model*	1 (Reference)	0.98 (0.73-1.32)	0.84 (0.65-1.08)	0.90 (0.70-1.15)	0.97 (0.75-1.27)	1.06 (0.83-1.34)
CVD mortality	Events, n (%)	350 (1.1)	15 (1.2)	24 (1.2)	34 (1.7)	24 (1.4)	37 (1.8)
(n = 40223)	Multivariable model*	1 (Reference)	0.77 (0.46-1.29)	0.77 (0.51-1.18)	1.04 (0.72-1.50)	0.87 (0.57-1.33)	1.07 (0.74-1.55)

Note: CVD, cardiovascular disease. one drink is equal to approximately 250 mL or 8.5 ounces.

* Multivariable model, estimates are hazard ratios (95% CIs) from multivariable Cox proportional hazard models adjusted for age (continuous), gender, Townsend deprivation index (continuous), education level (degree or no degree), ethnicity (white or other), smoking status (current, former, or never), pack-years of smoking (continuous), Overall health (poor, fair, good, or excellent), Basal metabolic rate (continuous), physical activity level (low, moderate, or high), body mass index (continuous), hypertension (yes or no), diabetes (yes or no), depression (yes or no), family history of CVD disease (yes or no), family history of cancer (yes or no), long-standing illness (yes or no), cholesterol-lowering drug use (yes or no), blood pressure drug use (yes or no), vitamin and mineral supplement (yes or no), and intake of energy, total sugar, fresh fruit, vegetables, red meat, processed meat, alcohol, coffee, milk, naturally sweet juices, sugar-sweetened beverages, and artificially sweetened beverages.

Table S18. Associations of tea consumption with all-cause and cause-specific mortality after additional adjustments to Alternative Healthy Eating Index 2010 (AHEI-2010).

			Tea consumers				
**Outcome**	**Events/Model**	**Nonconsumers**	**>0-1.5 drinks/d**	**>1.5-2.5 drinks/d**	**>2.5-3.5 drinks/d**	**>3.5-4.5 drinks/d**	**>4.5 drinks/d**
Unsweetened tea							
All-cause mortality	Events, n (%)	2286 (7.0)	946 (5.7)	1493 (5.4)	1593 (5.4)	1333 (5.2)	1752 (5.3)
(n = 165353)	Multivariable model*	1 (Reference)	0.9 (0.83-0.97)	0.82 (0.76-0.88)	0.82 (0.76-0.87)	0.78 (0.73-0.84)	0.80 (0.75-0.86)
Cancer mortality	Events, n (%)	1088 (3.6)	459 (3.0)	795 (3.2)	803 (3.0)	704 (3.0)	914 (3.0)
(n = 150677)	Multivariable model*	1 (Reference)	0.89 (0.80-1.00)	0.89 (0.81-0.98)	0.85 (0.77-0.93)	0.85 (0.77-0.94)	0.87 (0.78-0.95)
CVD mortality	Events, n (%)	440 (1.4)	170 (1.1)	261 (1.0)	285 (1.0)	209 (0.8)	284 (0.9)
(n = 158165)	Multivariable model*	1 (Reference)	0.87 (0.73-1.05)	0.78 (0.67-0.92)	0.81 (0.69-0.95)	0.69 (0.58-0.82)	0.74 (0.63-0.87)
Sugar-sweetened tea							
All-cause mortality	Events, n (%)	2286 (7.0)	192 (5.9)	326 (6.9)	310 (7.1)	251 (7.1)	339 (8.7)
(n = 52553)	Multivariable model*	1 (Reference)	0.81 (0.70-0.94)	0.95 (0.84-1.07)	0.91 (0.80-1.03)	0.85 (0.74-0.98)	0.87 (0.76-0.99)
Cancer mortality	Events, n (%)	1088 (3.6)	97 (3.3)	161 (3.7)	156 (3.9)	124 (3.8)	161 (4.5)
(n = 47921)	Multivariable model*	1 (Reference)	0.89 (0.72-1.10)	1.01 (0.85-1.20)	1.00 (0.84-1.20)	0.93 (0.76-1.14)	0.97 (0.80-1.17)
CVD mortality	Events, n (%)	440 (1.4)	43 (1.4)	61 (1.3)	57 (1.4)	44 (1.3)	67 (1.8)
(n = 50348)	Multivariable model*	1 (Reference)	0.95 (0.69-1.31)	0.94 (0.71-1.24)	0.9 (0.67-1.20)	0.8 (0.58-1.12)	0.89 (0.66-1.20)
Artificially sweetened tea							
All-cause mortality	Events, n (%)	2286 (7.0)	125 (8.4)	187 (8.3)	204 (9.2)	164 (8.6)	217 (9.7)
(n = 42918)	Multivariable model*	1 (Reference)	0.91 (0.76-1.09)	0.87 (0.75-1.01)	0.91 (0.79-1.06)	0.88 (0.75-1.03)	0.90 (0.77-1.04)
Cancer mortality	Events, n (%)	1088 (3.6)	55 (4.0)	79 (3.9)	90 (4.5)	81 (4.6)	100 (4.9)
(n = 39109)	Multivariable model*	1 (Reference)	0.88 (0.67-1.16)	0.86 (0.68-1.08)	0.94 (0.75-1.17)	0.98 (0.78-1.24)	0.96 (0.77-1.19)
CVD mortality	Events, n (%)	440 (1.4)	28 (2.0)	42 (2.0)	49 (2.3)	28 (1.5)	45 (2.1)
(n = 41072)	Multivariable model*	1 (Reference)	0.96 (0.65-1.41)	0.94 (0.68-1.29)	1.05 (0.78-1.43)	0.73 (0.49-1.08)	0.92 (0.66-1.28)

Note: CVD, cardiovascular disease; AHEI-2010, Alternative Healthy Eating Index 2010. one drink is equal to approximately 250 mL or 8.5 ounces.

* Multivariable model, estimates are hazard ratios (95% CIs) from multivariable Cox proportional hazard models adjusted for age (continuous), gender, Townsend deprivation index (continuous), education level (degree or no degree), ethnicity (white or other), smoking status (current, former, or never), pack-years of smoking (continuous), Overall health (poor, fair, good, or excellent), Basal metabolic rate (continuous), physical activity level (low, moderate, or high), body mass index (continuous), hypertension (yes or no), diabetes (yes or no), depression (yes or no), family history of CVD disease (yes or no), family history of cancer (yes or no), long-standing illness (yes or no), cholesterol-lowering drug use (yes or no), blood pressure drug use (yes or no), vitamin and mineral supplement (yes or no), and intake of total sugar, coffee, milk, and AHEI-2010.

Table S19. Associations of tea consumption with all-cause and cause-specific mortality after additional adjustments to the number of long-term chronic conditions.

Outcome	Events/Model	Nonconsumers	Tea consumers				
			>0-1.5 drinks/d	>1.5-2.5 drinks/d	>2.5-3.5 drinks/d	>3.5-4.5 drinks/d	>4.5 drinks/d
**Unsweetened tea**							
All-cause mortality	Events, n (%)	2286 (7.0)	946 (5.7)	1493 (5.4)	1593 (5.4)	1333 (5.2)	1752 (5.3)
(n = 165503)	Multivariable model*	1 (Reference)	0.89 (0.83-0.97)	0.81 (0.76-0.87)	0.82 (0.77-0.88)	0.78 (0.73-0.84)	0.81 (0.76-0.87)
Cancer mortality	Events, n (%)	941 (0.03)	401 (3.1)	668 (2.6)	658 (2.6)	606 (2.5)	774 (2.6)
(n = 150807)	Multivariable model*	1 (Reference)	0.89 (0.79-1)	0.86 (0.78-0.96)	0.81 (0.72-0.9)	0.86 (0.77-0.96)	0.87 (0.78-0.97)
CVD mortality	Events, n (%)	350 (1.1)	147 (0.9)	213 (0.8)	244 (0.9)	179 (0.7)	228 (0.7)
(n = 158877)	Multivariable model*	1 (Reference)	0.91 (0.75-1.1)	0.76 (0.64-0.91)	0.83 (0.7-0.99)	0.71 (0.59-0.87)	0.74 (0.61-0.89)
**Sugar-sweetened tea**							
All-cause mortality	Events, n (%)	2286 (7.0)	192 (5.9)	326 (6.9)	310 (7.1)	251 (7.1)	339 (8.6)
(n = 52597)	Multivariable model*	1 (Reference)	0.79 (0.68-0.92)	0.91 (0.81-1.03)	0.88 (0.78-1)	0.83 (0.72-0.96)	0.85 (0.74-0.97)
Cancer mortality	Events, n (%)	941 (3.1)	80 (2.7)	144 (3.3)	139 (3.4)	108 (3.4)	136 (3.8)
(n = 48303)	Multivariable model*	1 (Reference)	0.84 (0.67-1.06)	1.02 (0.85-1.23)	1 (0.83-1.22)	0.96 (0.77-1.19)	0.93 (0.75-1.15)
CVD mortality	Events, n (%)	350 (1.1)	35 (1.1)	49 (1.1)	47 (1.1)	35 (1.1)	54 (1.5)
(n = 49977)	Multivariable model*	1 (Reference)	0.88 (0.62-1.26)	0.88 (0.65-1.21)	0.87 (0.63-1.2)	0.78 (0.54-1.14)	0.9 (0.65-1.26)
**Artificially sweetened tea**							
All-cause mortality	Events, n (%)	2286 (7.0)	125 (8.4)	187 (8.3)	204 (9.2)	164 (8.6)	217 (9.7)
(n = 42951)	Multivariable model*	1 (Reference)	0.92 (0.77-1.11)	0.91 (0.78-1.05)	0.94 (0.81-1.09)	0.9 (0.76-1.06)	0.95 (0.81-1.1)
Cancer mortality	Events, n (%)	941 (3.1)	54 (3.9)	68 (3.3)	71 (3.6)	63 (3.7)	91 (4.5)
(n = 39305)	Multivariable model*	1 (Reference)	1.03 (0.78-1.35)	0.86 (0.67-1.1)	0.9 (0.7-1.16)	0.97 (0.74-1.26)	1.1 (0.87-1.39)
CVD mortality	Events, n (%)	350 (1.1)	15 (1.1)	25 (1.2)	36 (1.8)	24 (1.4)	37 (1.8)
(n = 40562)	Multivariable model*	1 (Reference)	0.71 (0.42-1.2)	0.79 (0.52-1.19)	1.06 (0.75-1.52)	0.85 (0.55-1.3)	1.06 (0.73-1.53)

Note: CVD, cardiovascular disease. one drink is equal to approximately 250 mL or 8.5 ounces.

* Multivariable model, estimates are hazard ratios (95% CIs) from multivariable Cox proportional hazard models adjusted for age (continuous), gender, Townsend deprivation index (continuous), education level (degree or no degree), ethnicity (white or other), smoking status (current, former, or never), pack-years of smoking (continuous), Basal metabolic rate (continuous), physical activity level (low, moderate, or high), body mass index (continuous), family history of CVD disease (yes or no), family history of cancer (yes or no), vitamin and mineral supplement (yes or no), number of long-term chronic conditions (none, one, two, three and more), and intake of energy, total sugar, fresh fruit, vegetables, red meat, processed meat, alcohol, coffee, milk, naturally sweet juices, sugar-sweetened beverages, and artificially sweetened beverages.

Table S20. The E-values of tea consumption with all-cause mortality and cause-specific mortality, E-value (LCL).

Outcome	Nonconsumers	Tea consumers				
		0-1.5 drinks/d	1.5-2.5 drinks/d	2.5-3.5 drinks/d	3.5-4.5 drinks/d	>4.5 drinks/d
**Unsweetened tea**						
All-cause mortality	1 (Reference)	1.39 (1.11)	1.70 (1.50)	1.67 (1.46)	1.81 (1.60)	1.70 (1.50)
Cancer mortality	1 (Reference)	1.46 (1.11)	1.56 (1.25)	1.77 (1.46)	1.60 (1.21)	1.56 (1.21)
CVD mortality	1 (Reference)	1.35 (1.00)	1.88 (1.36)	1.63 (1.00)	2.08 (1.50)	2.00 (1.43)
**Sugar-sweetened tea**						
All-cause mortality	1 (Reference)	1.77 (1.32)	1.29 (1.00)	1.43 (1.00)	1.63 (1.16)	1.56 (1.11)
Cancer mortality	1 (Reference)	1.67 (1.00)	1.16 (1.00)	1.11 (1.00)	1.32 (1.00)	1.36 (1.00)
CVD mortality	1 (Reference)	1.39 (1.00)	1.32 (1.00)	1.43 (1.00)	1.70 (1.00)	1.29 (1.00)
**Artificially sweetened tea**						
All-cause mortality	1 (Reference)	1.43 (1.00)	1.50 (1.00)	1.32 (1.00)	1.46 (1.00)	1.32 (1.00)
Cancer mortality	1 (Reference)	1.11 (1.00)	1.67 (1,00)	1.50 (1.00)	1.25 (1.00)	1.40 (1.00)
CVD mortality	1 (Reference)	2.17 (1.00)	1.88 (1.00)	1.34 (1.00)	1.63 (1.00)	1.28 (1.00)

Note: E-value (LCL) was calculated for sensitivity analysis, LCL, lower control limit.
